# Termination factor Rho mediates transcriptional reprogramming of *Bacillus subtilis* stationary phase

**DOI:** 10.1371/journal.pgen.1010618

**Published:** 2023-02-03

**Authors:** Vladimir Bidnenko, Pierre Nicolas, Cyprien Guérin, Sandra Dérozier, Arnaud Chastanet, Julien Dairou, Yulia Redko-Hamel, Matthieu Jules, Elena Bidnenko

**Affiliations:** 1 Micalis Institute, INRAE, AgroParisTech, Université Paris-Saclay, Jouy-en-Josas, France; 2 MaIAGE, INRAE, Université Paris-Saclay, Jouy-en-Josas, France; 3 Laboratoire de Chimie et Biochimie Pharmacologiques et Toxicologiques, CNRS, UMR 8601, Université de Paris, Paris, France; Tufts University School of Medicine, UNITED STATES

## Abstract

Transcription termination factor Rho is known for its ubiquitous role in suppression of pervasive, mostly antisense, transcription. In the model Gram-positive bacterium *Bacillus subtilis*, de-repression of pervasive transcription by inactivation of *rho* revealed the role of Rho in the regulation of post-exponential differentiation programs. To identify other aspects of the regulatory role of Rho during adaptation to starvation, we have constructed a *B*. *subtilis* strain (Rho^+^) that expresses *rho* at a relatively stable high level in order to compensate for its decrease in the wild-type cells entering stationary phase. The RNAseq analysis of Rho^+^, WT and Δ*rho* strains (expression profiles can be visualized at http://genoscapist.migale.inrae.fr/seb_rho/) shows that Rho over-production enhances the termination efficiency of Rho-sensitive terminators, thus reducing transcriptional read-through and antisense transcription genome-wide. Moreover, the Rho^+^ strain exhibits global alterations of sense transcription with the most significant changes observed for the AbrB, CodY, and stringent response regulons, forming the pathways governing the transition to stationary phase. Subsequent physiological analyses demonstrated that maintaining *rho* expression at a stable elevated level modifies stationary phase-specific physiology of *B*. *subtilis* cells, weakens stringent response, and thereby negatively affects the cellular adaptation to nutrient limitations and other stresses, and blocks the development of genetic competence and sporulation. These results highlight the Rho-specific termination of transcription as a novel element controlling stationary phase. The release of this control by decreasing Rho levels during the transition to stationary phase appears crucial for the functionality of complex gene networks ensuring *B*. *subtilis* survival in stationary phase.

## Author summary

The Gram-positive soil-dwelling bacterium *Bacillus subtilis* is well known for its ability to adapt to changing and often unfavorable environmental conditions. To improve the chances of survival, *B*. *subtilis* employs various strategies that range from global modifications of cellular metabolism that are characteristic to stationary phase and allowing starvation resistance, to alternative programs of cellular differentiation, including the formation of dormant and highly resistant spores as an ultimate survival option. All these processes rely on the functioning of complex and interconnected gene networks, which are controlled by several well-known global transcription regulators acting at the level of transcription initiation. Here we show that the termination factor Rho, responsible for the main factor-dependent pathway of transcription termination and the major inhibitor of antisense transcription in bacteria, is intricately involved in the control of adaptation of *B*. *subtilis* cells to stationary phase. Notably, maintaining Rho at a constant level during exponential growth and stationary phase triggers transcriptional reprogramming and modifies the stationary-phase physiology of *B*. *subtilis* cells to such an extent that it negatively affects cellular adaptation to nutrient limitation and cell-fate decision-making. Therefore, the transcription termination factor Rho imposes a new layer of control over stationary phase and adaptive strategies in *B*. *subtilis*.

## Introduction

Transcription termination is crucial for the precise regulation of gene expression and maintenance of genome integrity in all living organisms [1–4]. In bacteria, transcription termination is achieved by two main mechanisms: intrinsic, which is associated with specific sequence forming an RNA terminator hairpin essential for disruption of the transcription elongation complex (TEC), and factor-dependent, which relies mostly on the activity of an RNA helicase–translocase, the transcription termination factor Rho [1,5–9]. Specific interaction of Rho with RNA polymerase destabilizes active TEC leading to its subsequent dissociation and release of a transcript [10–14]. Although historically the intrinsic termination pathway has long been considered factor-independent, Rho has been shown to act on some suboptimal intrinsic terminators thereby preventing their read-through [14–16]. Similarly, efficient intrinsic termination often requires activity of transcription elongation factors NusA and NusG [6,17–22]. Both proteins have been shown to contribute to some Rho-dependent termination events *in vitro* and *in vivo* [10,12,13,23–28]. The recent studies have shown that combined activities of NusA, NusG, and Rho stimulate approximately 97% of all intrinsic terminators in the model Gram-positive bacterium *Bacillus subtilis* [reviewed in 28]. Considering the crucial role of the transcription factors in the control of both transcription termination pathways, it is not surprising that inactivation or depletion of any of these three proteins affects gene expression [15,21–23,26–30].

In line with its molecular function, Rho is recognized as a major factor controlling pervasive, mostly antisense, transcription in different bacterial species [15,30–32]. Moreover, deletion of the *rho* gene or partial inhibition of Rho activity has been shown to alter the expression of protein-coding genes and various non-coding RNAs (ncRNAs) by a combination of direct (transcription read-through of Rho-controlled terminators) and different indirect effects (e.g., resulting from regulatory cascades initiated by direct events), thus affecting diverse physiological processes [26,31–35]. However, the biological significance of Rho-controlled antisense RNAs (asRNAs) and ncRNAs, as well as the mechanisms underlying the involvement of Rho-mediated transcription termination in bacterial physiology, are still not well understood.

By using a *B*. *subtilis Δrho* mutant, we have shown that several Rho-controlled sense and antisense transcripts contribute to the coordinated regulation of mutually exclusive cell differentiation programs (motility, biofilm formation, and sporulation) controlled by the master regulator Spo0A [26]. In particular, we demonstrated that deletion of the *rho* gene prevents Rho-dependent intragenic termination of the *kinB* transcript encoding the sensor kinase KinB, thereby increasing cellular levels of the active phosphorylated form of Spo0A (Spo0A~P) to a threshold triggering sporulation. Thus, Rho inactivation stimulates sporulation, an ultimate survival option of *B*. *subtilis* cells entering stationary phase due to nutrient deprivation [26]. In the human pathogens *Salmonella enterica*, *Staphylococcus aureus*, *Mycobacterium tuberculosis*, and *Clostridioides difficile*, inhibition of Rho activity induces the expression of virulence factors essential for the successful host colonization and infection [27,29,32,33,35]. Likewise, Rho inactivation affects expression of genes involved in cellular differentiation, colonization and pathogenesis in *Bacillus thuringiensis* [34].

Overall, the data accumulated on Rho-deficient cells indicate that in *B*. *subtilis* and other Gram-positive bacteria, Rho plays an important role in the regulation of different phenomena associated with stationary phase. It is also noteworthy that in *B*. *subtilis* and *S*. *aureus*, the expression of *rho* decreases in stationary phase [15,26,30].

Transition to stationary phase is characterized by a slowdown of macromolecular synthesis, reorientation of the cellular metabolism towards alternative metabolic pathways and the activation of stress response networks [36]. In *B*. *subtilis*, two other transcriptional regulators sensing environmental and intracellular metabolic status, AbrB and CodY, drive the reprogramming of metabolism and cellular differentiation in addition to Spo0A [37]. During exponential growth, AbrB suppresses the expression of over two hundred genes that are switched ON upon the programmed depletion of AbrB in cells entering stationary phase [38–40]. AbrB is an essential component of the regulatory networks governing the initiation of sporulation and development of genetic competence [41,42]. Expression of the *abrB* gene is repressed by Spo0A~P, which also controls AbrB DNA binding activity [39,43,44]. In addition, AbrB abundancy is negatively controlled at the post-transcriptional level by ncRNA, RnaC [45]. The pleiotropic regulator CodY represses expression of the numerous genes required for adaptation to nutrient limitation [46,47]. This repression is released to activate the alternative nutrient acquisition pathways when cells enter into stationary phase [46–49]. CodY is also implicated in the control of genetic competence and sporulation [50,51]. CodY modulates its own DNA-binding affinity by sensing specific metabolites: branched-chain amino acids (BCAA) and the nucleoside triphosphate GTP. In the absence of any of these ligands, the ability of CodY to bind DNA is impaired [48,52]. In that way, activity of CodY is linked to stringent response (SR), a widespread stress resistance mechanism essential for stationary phase survival [53–55]. The SR is characterized by the synthesis of the alarmone guanosine-(penta)tetra-phosphate ((p)ppGpp), mainly provided by a bifunctional synthetase/hydrolase Rel sensing starved ribosomes [56–58]. In *B*. *subtilis*, (p)ppGpp modifies genome-wide transcription indirectly, by causing a decrease in GTP levels due to the inhibition of activity of GTP-synthesizing enzymes and consumption of GTP during synthesis of (p)ppGpp [59–62]. A decrease in the GTP levels causes de-repression of the CodY regulon, negatively affects transcription from promoters of stable RNA synthesis genes (e. g., genes involved in the ribosome biogenesis) and re-directs RNA polymerase from these GTP-initiating promoters to promoters of biosynthetic genes [63–65]. In addition, (p)ppGpp represses DNA replication [66] and inhibits protein synthesis [67–69]. Furthermore, the transitional increase of (p)ppGpp contributes to the induction of genetic competence and sporulation [51,59,70,71].

As mentioned above, the levels of *rho* mRNA and Rho protein decrease in wild-type *B*. *subtilis* cells upon entry into stationary phase, which is reminiscent to that observed for the transition state regulator AbrB [15,26,39]. By analogy with AbrB, this phase-dependent reduction in Rho amount suggests that, in addition to cell fate determination [26], Rho may be involved in other regulatory networks that promote early stages of cellular adaptation to starvation. Such hypothetical Rho activity could not be detected using the Δ*rho* mutant, which mimics stationary phase-dependent decrease in the *rho* expression to its extreme degree and in which, due to the absence of Rho, the regulation of the respective gene networks is altered in favor of sporulation. Therefore, we assumed that if some stationary phase-specific modifications of *B*. *subtilis* transcription and physiology were linked to a decrease in Rho content, they would be altered in cells expressing Rho at a higher level. Following this hypothesis, we engineered and analyzed a *B*. *subtilis* strain, which expressed Rho at an elevated stable level throughout exponential growth and early stationary phase.

We show that maintaining Rho at a high level causes a global inhibition of pervasive antisense transcription, including transcriptional noise, and induces genome-wide alterations of gene expression. These changes are associated with a weakening of the SR and a reprogramming of starvation-specific developmental programs driven by the key transcriptional regulators AbrB, CodY and Spo0A.

These findings provide new insights into the role of the transcription termination factor Rho in the physiology of *B*. *subtilis* and suggest that *in fine* regulation of the Rho-controlled transcription is crucial for the functionality of the complex gene networks governing the stationary-phase adaptation in this model Gram-positive bacterium.

## Results

### Heterogenic system for *rho* over-expression

**Fig 1:**
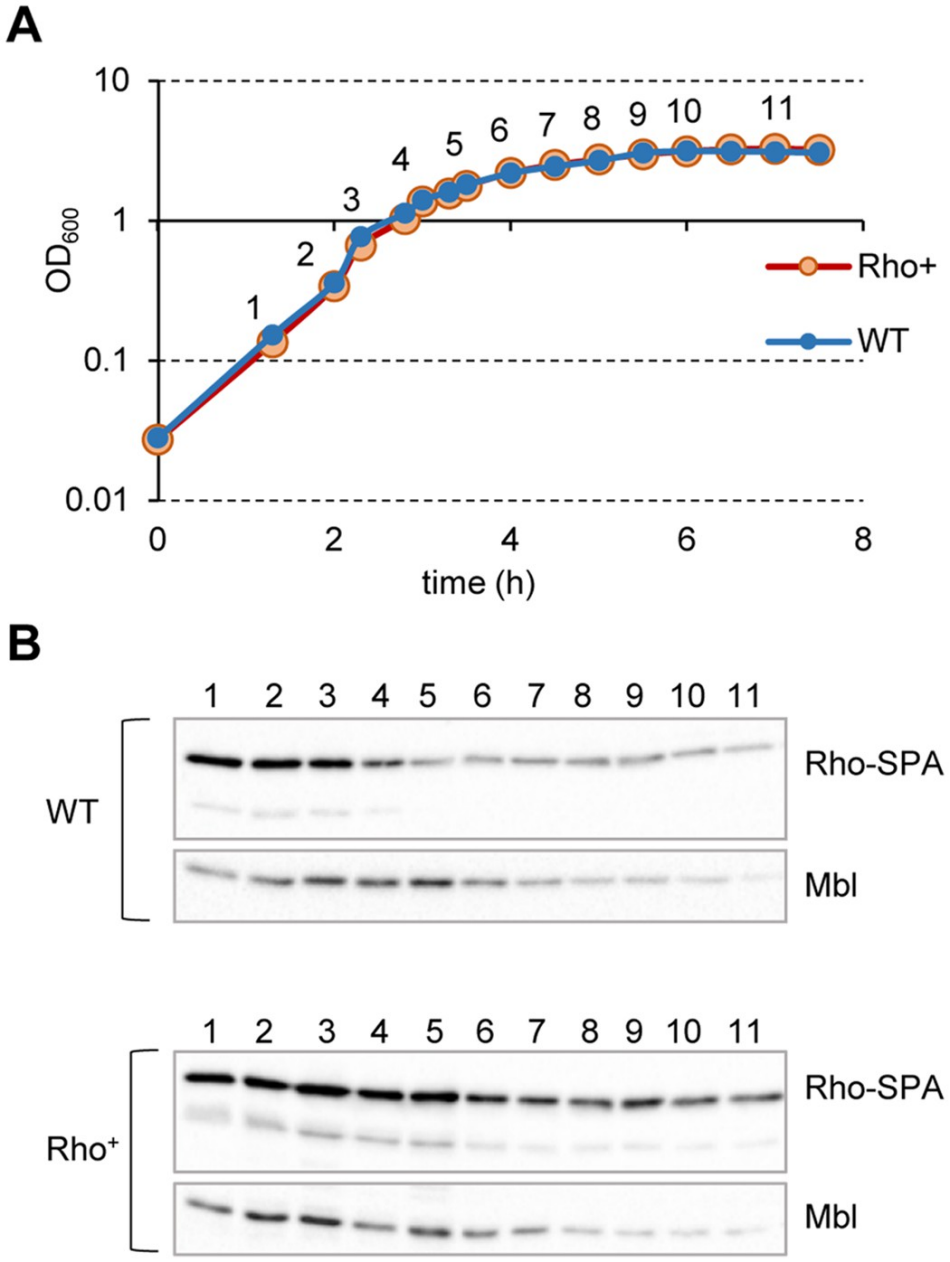
Heterogenic expression of the transcription termination factor Rho. *B*. *subtilis* cells expressing SPA-tagged Rho protein from natural (WT) and P*veg* (Rho^+^) promoters were grown in LB medium (**A**) and analyzed for Rho protein content at the indicated time points by immunoblotting with ANTI-FLAG M2 monoclonal antibodies (Rho-SPA; **B**). Equal amounts of total protein extracts were loaded onto the gel according to Bradford assay; samples’ equilibrium between the strains at each time-point and the quality of transfer were controlled by visualization of Mbl protein using specific anti-Mbl antibodies (Mbl; **B**). Note that the Mbl levels decrease in stationary phase.

To evaluate the impact of the increased Rho levels on stationary phase-specific phenomena, we engineered a system that would maintain relatively stable Rho amounts during exponential growth and stationary phase. For this purpose, we disconnected *rho* expression from any regulatory circuits acting at its natural locus [72] by placing an additional copy of the *rho* gene under the control of *B*. *subtilis* constitutive promoter P_*veg*_ and ribosome binding site of the *tagD* gene [73–75]. The engineered *rho* expression unit was inserted into the *amyE* locus of the chromosome of wild-type strain BSB1 (hereinafter, WT), for which transcriptome-based annotation is available [15]. The growth rate of the resulting strain (Rho^+^) in rich LB medium was not affected by the ectopic *rho* expression over an extended period (~10 h) (Fig 1A).

To compare dynamics of Rho expression in WT and Rho^+^ cells, we constructed and analyzed by immunoblotting the sibling strains, which express, at natural and ectopic locations, the SPA-tagged Rho protein [26]. As shown in Fig 1B, both strains exhibited a decrease of Rho-SPA amounts during stationary phase. However, Rho-SPA was present at a higher level and disappeared at a slower rate in Rho^+^ than in WT cells. This was most probably due to the inherently strong activity of the constitutive P_*veg*_ promoter weakening during stationary phase [74].

Further, using mass spectrometry analysis of WT and Rho^+^ cells expressing the non-tagged Rho (see below), we showed that the amount of Rho protein was ~3 fold higher in Rho^+^ compared to WT during exponential growth. In stationary phase, the Rho level dropped around 2.6-fold in WT, but it remained stable in Rho^+^ cells (S1 Table).

We concluded that using the heterogenic expression system assures a steady high level of Rho expression throughout exponential growth and up to the early stationary phase.

### The Rho^+^ strain exhibits sporulation-deficient phenotype

**Fig 2:**
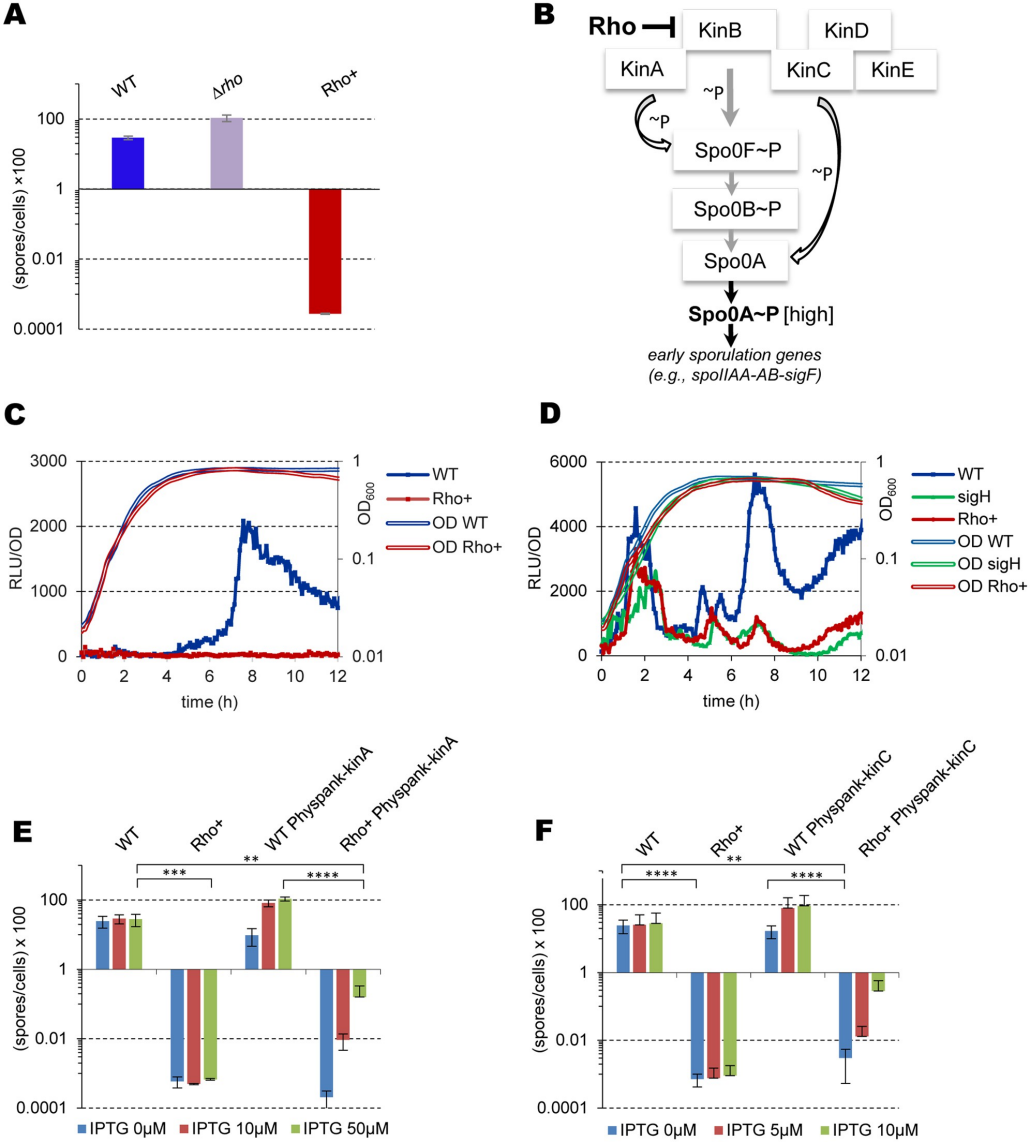
The Rho^+^ strain exhibits sporulation-negative phenotype. (**A**) Sporulation efficiency of *B*. *subtilis* WT, Δ*rho* and Rho^+^ cells. Cells inoculated at OD_600_ 0.025 were grown in DS medium at 37° for 20 hours and analyzed for heat-resistant spores as described in Materials and Methods. Sporulation efficiency was estimated as proportion of viable cells in the heated and unheated cultures. Plotted are the mean values from four independent experiments with three biological replicas for each strain with standard deviation SD (bar-headed lines). (**B**) Schematics of *the* multicomponent Spo0A phosphorelay. Only the key elements relevant to this study are shown. Phosphoryl groups are transferred from sensor protein kinases (KinA-E) to Spo0F, Spo0B, and ultimately to Spo0A. Sporulation is triggered when the level of Spo0A∼P reaches a high threshold level. The bar-headed line indicates negative Rho-mediated regulation of KinB expression. (**C**) Kinetics of luciferase expression from the promoter of early sporulation gene *spoIIAA* in *B*. *subtilis* WT (blue line) and Rho^+^ (red line) cells induced for sporulation. (**D**) Kinetics of luciferase expression from the promoters of *spo0A* gene in WT (blue line), Rho^+^ (red line) and *sigH* mutant (green line) cells induced for sporulation. In **C** and **D**, cells bearing transcriptional fusions *spoIIAA-luc* and *spo0A-luc*, respectively, were grown in DS medium and analyzed for luciferase activity at five-minute intervals in a multimode microplate reader as described in Materials and Methods. For each strain, plotted are the mean values of luminescence readings corrected for OD_600_ from four independent cultures analyzed simultaneously (solid lines with symbols) and characteristic growth curves (double-lined) measured by OD_600_. The experiments were reproduced at least three times. The results from the representative experiments are shown. (**E** and **F**) Synthetic over-production of sensor histidine kinases KinA or KinC does not rescue sporulation-negative phenotype of Rho^+^ cells. Sporulation efficiency of *B*. *subtilis* WT and Rho^+^ strains and their respective derivatives expressing *kinA* (**E**) and *kinC* (**F**) genes under control of the IPTG-inducible promoter. Cells were inoculated at OD_600_ 0.025 in DS medium containing IPTG at the indicated concentrations and grown at 37° during 20 hours; sporulation efficiency was analyzed as described above (Fig 2A) and in Materials and Methods. Plotted are the mean values from three independent experiments with three biological replicas of each strain with standard deviation SD (bar-headed lines). Statistical significance was estimated with a two-tailed t-test. The displayed *p*-values are as follows: ****, p ≤ 0.0001; ***, p ≤ 0.001; **, p ≤ 0.01.

We initiated the analysis of physiological effects of a steady high Rho content by assessment of the sporulation capacity of Rho^+^ cells. The WT, Δ*rho* mutant and Rho^+^ cells were compared for the ability to form heat-resistant spores in the sporulation-inducing DS medium (Materials and methods). Depending on the experiment, 20% to 40% of the WT cells developed spores under the used conditions, while *rho* deletion increased the sporulation rate to almost 100% as previously reported (Fig 2A, [26]). In contrast, the efficiency of spore formation by the Rho^+^ strain was reduced up to 10^−5^. The rare spores isolated from Rho^+^ cultures appeared to be suppressor mutants able to form spores with a near-WT efficiency (S2 Table). Analysis of several independent sporulation-proficient Rho^+^ suppressors revealed different point mutations within the *rho* coding sequence of the ectopic *rho* expression unit (S1 Fig and S2 Table). This reaffirms the determining role of Rho in the sporulation-deficient phenotype of Rho^+^ cells.

Considering that Rho affects activity of the Spo0A phosphorelay (Fig 2B) [26], strong inhibition of sporulation in Rho^+^ cells suggested significant reduction of active Spo0A~P.

To test this hypothesis, we analyzed expression of the *spoIIAA-AB-sigF* operon using the transcriptional fusion of its promoter to the firefly luciferase gene *luc* (P_*spoIIAA*_*-luc*) [26]. Expression of the *spoIIAA-AB-sigF* operon is activated at a high threshold level of Spo0A~P (Fig 2B) and depends on alternative SigH factor, which itself is under positive indirect control of Spo0A~P by disabling AbrB repressor [44,76–78]. As shown in Fig 2C, whereas in WT cells the P_*spoIIAA*_ promoter was switched ON roughly three hours after the entry into stationary phase, no expression could be detected in Rho^+^ cells, suggesting that cells failed to accumulate sufficient amount of Spo0A~P.

Accumulating Spo0A~P during the transition to stationary phase increases *spo0A* expression via several positive feedback loops [79]. Thus, we analyzed the expression of the *spo0A* gene. The reporter P_*spo0A*_-*luc* fusion was integrated into the natural *spo0A* locus and monitors activity of both vegetative SigA-dependent (Pv) and sporulation SigH/Spo0A-controlled (Ps) promoters [26,71,80–82]. During exponential growth, the P_*spo0A*_-*luc* expression was similar in WT and Rho^+^ cells suggesting that Pv promoter was not affected by Rho (Fig 2D). In contrast, two hours after the entry into stationary phase, P_*spo0A*_-*luc* activity greatly increased in WT cells, but remained low in Rho^+^ cells. We noticed that expression kinetics of the P_*spo0A*_*-luc* in Rho^+^ cells was similar to that observed in the *sigH* mutant, in which the activity of Ps promoter is abolished (Fig 2D); [81,82]. This suggests that promoter Ps of the *spo0A* gene was inactive in Rho^+^ cells either due to the Spo0A~P level lower than required for promoter’s activation or, not mutually exclusive, due to the low activity of SigH.

Thus, over-production of Rho causes a sporulation-negative phenotype of *B*. *subtilis* cells associated with down-regulation of *spo0*A gene expression, which, according to our previous results, may have been due to an effective repression of the Spo0A phosphorelay [26].

### Synthetic over-production of sensor histidine kinases KinA or KinC does not rescue the sporulation-deficient phenotype of the Rho^+^ strain

To investigate whether the sporulation-negative phenotype of Rho^+^ cells was solely due to a low activity of the Spo0A phosphorelay, we attempted to boost Spo0A phosphorylation (Fig 2B).

To this end, we used a system over-expressing the major sporulation kinase, KinA, from an IPTG-inducible promoter (P_*hyspank*_-*kinA*) [80]. As shown in Fig 2E, addition of IPTG at 10μM and 50μM (concentrations shown to induce the *kinA* gene to a saturation level and stimulate sporulaton during exponential growth [80,83]), triggered formation of the heat-resistant spores in almost 100% of WT cells. The over-expression of KinA in Rho^+^ cells also increased the sporulation frequency ~10^3^-fold, which remained, however, much below the sporulation level observed in WT cells (Fig 2E).

To relieve the accumulation of Spo0A~P from the control of phosphorelay, we used a P_*hyspank*_-*kinC* system over-producing the sensor kinase KinC, known to transfer phosphate directly to Spo0A [80,84,85]. As shown in Fig 2F, induction of KinC expression at IPTG concentrations of 5 to 10 μM, previously shown to be optimal for proper activation of Spo0A [86], stimulated sporulation in WT cells to maximal levels (~ 93%), but resulted only in a partial restoration of sporulation efficiency (0.01% and 0.3% at 5 and 10 μM IPTG, respectively) in the Rho^+^ strain.

To learn more about the sporulation defect of Rho^+^ cells, we performed microscopy analysis of cells differentially expressing *kinA*, which were synchronously induced to sporulate by resuspension of the actively growing cultures in a poor salt medium (Materials and methods). Three hours after resuspension, Rho^+^ and Rho^+^ P_*hyspank*_-*kinA* cells appeared significantly longer than their WT relatives, which suggests that cells over-expressing Rho react differently to the nutrient shift. At this time point, ~18.7% and ~26.6% of the WT and the non-induced WT P_*hyspank*_-*kinA* cells, respectively, contained forespores (S2 Fig). The proportion of WT P_*hyspank*_-*kinA* cells with forespores increased up to 89% after KinA induction by IPTG (S2 Fig). In contrast, no forespores were observed in the Rho^+^ and the non-induced Rho^+^ P_*hyspank*_-*kinA* cultures. Induction of *kinA* stimulated formation of forespores in ~10.7% of Rho^+^ P_*hyspank*_-*kinA* cells, which is 8-fold lower than the induced WT P_*hyspank*_-*kinA* and about twice lower than in the WT cells.

Overall, we concluded that over-production of sensor kinases ensuring consequent increase of Spo0A phosphorylation either directly (KinC) or via the phosphorelay (KinA) cannot fully suppress the sporulation-negative phenotype of Rho^+^ cells. This suggests that Rho negatively affects sporulation not only at the level of Spo0A phosphorylation, but also at other stage(s).

### The Rho^+^ strain exhibits competence-deficient phenotype

**Fig 3:**
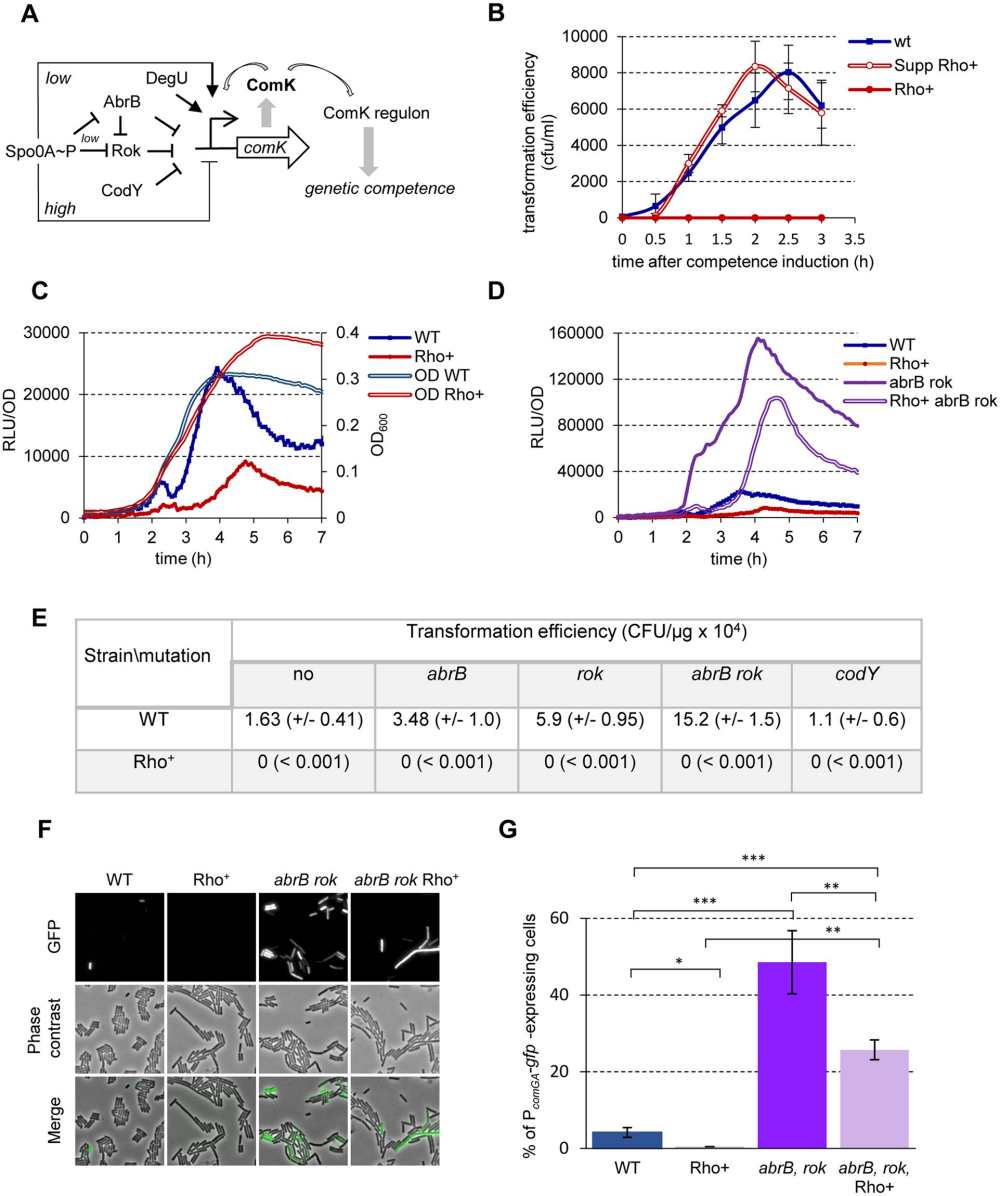
*B*. *subtilis* Rho^+^ exhibits competence–negative phenotype. **(A)** Schematics of P_*comK*_ regulation in *B*. *subtilis* cells. The promoter of the *comK* gene (P_*comK*_) encoding the master regulator of competence is regulated by five transcription factors: Spo0A, Rok, AbrB, CodY, and DegU, and by ComK itself. The gradual increase in Spo0A∼P first induces the P_*comK*_ promoter activity directly (topmost arrow) and by antagonizing the transcriptional repressors AbrB and Rok, but represses it when concentration of Spo0A~P reaches a high threshold level (bottommost bar-headed line). Along with action on the P_*comK*_, AbrB represses the promoter of *rok*. ComK activates its own expression by direct binding to P_*comK*_ assisted by the response regulator DegU. Eventually, an increase of ComK leads to the activation of the expression of DNA-binding, -uptake and -recombination genes that determines development of the competence state. Arrows and bar-headed lines represent positive and negative effects, respectively. **(B)** Kinetics of competence development in *B*. *subtilis* WT (blue line), Rho^+^ (red line) and Rho^+^_Q146Stop_ (red double-line) strains. Cells grown in a defined rich medium SpC to stationary phase were transferred to competence medium (T0) and tested for transformation by homologous genomic DNA over three hours as described in Materials and Methods. The experiment included three biological replicas of each strain and was reproduced three times. Plotted are the mean values and SD from a representative experiment. **(C)** Kinetics of luciferase expression from the promoter of *comK* gene in *B*. *subtilis* WT (blue line) and Rho^+^ (red line) cells. Cells bearing the P_*comK*_*-luc* transcription fusion were grown in competence medium and analyzed for luciferase activity as described in Materials and Methods. Plotted are the mean values of luminescence readings corrected for OD from four independent cultures of each strain analyzed simultaneously. The double-lined curves depict characteristic growth kinetics of cells measured by OD_600_. (**D**) Kinetics of luciferase expression in *B*. *subtilis* mutant strains: *abrB rok* P_*comK-luc*_ (purple line) and Rho^+^ *abrB rok* P_*comK-luc*_ (purple double-line). The mutants were analyzed in parallel with the parental strains WT P_*comK-luc*_ (blue line) and Rho^+^ P_*comK-luc*_ (red line). For each strain, data acquisition and processing were performed as in (C). Each strain was analyzed at least three times. The results of representative experiments are shown. (**E**) Inactivation of the known repressors of *comK* does not rescue competence–negative phenotype of Rho^+^ cells. *B*. *subtilis* WT and Rho^+^ strains and their respective derivatives carrying single mutations in the *abrB*, *rok*, *codY* genes or double *abrB rok* mutation were transformed by donor genomic DNA after two hours of growth in competence medium as described in Materials and Methods. Shown are the mean values with SD (in parentheses) from two independent experiments each incorporating three biological replicas of each strain. (**F and G)** De-repression of *comK* leads to the activation of the late competence genes in Rho^+^ cells. (**F**) The WT P_*comGA*_-*gfp*, Rho^+^ P_*comGA*_-*gfp* strains and their *abrB rok* mutant derivatives were grown in competence medium up to stationary phase (OD_600_ 1.5). Cells were sampled two hours after entering stationary phase and analyzed by fluorescence microscopy for the activity of P_*comGA*_-*gfp* reporter in two replicas. (**G)** The proportion of GFP-expressing cells was determined by manual counting of a minimum 600 cells per strain and per replica. The experiment was reproduced twice. Plotted are the mean values and SD from a representative experiment. Statistical significance was estimated with a two-tailed t-test. The displayed p-values are as follows: ***, p ≤ 0.001; **, p ≤ 0.01; *, 0.01 ≤ p ≤ 0.05.

The intermediate Spo0A~P level, which increases transiently during the late exponential growth without the activation of the sporulation-specific *spo0A* promoter Ps, was shown to be crucial for development of genetic competence (Fig 3A) [87,88].

We found that, contrary to WT, the Rho^+^ strain was not transformable using a standard two-step transformation procedure (Materials and methods). To better characterize this phenotype, we followed the kinetics of competence development by transforming cells by genomic or plasmid DNA at different time after transfer to a competence-inducing medium. As shown in Fig 3B and S3 Fig, while the efficiency of transformation of WT cells gradually increased during ~2.5 hours of growth in competence medium, Rho^+^ cells remained transformation-deficient all over the experiment. The primary role of Rho in the competence–negative phenotype of Rho^+^ cells was confirmed by the WT-like transformation efficiency of the Rho^+^_Q146Stop_ suppressor mutant (Fig 3B).

To determine whether the competence-negative phenotype of Rho^+^ cells is caused by low expression of the *comK* gene encoding the master regulator of competence [89], we followed the activity of the *comK* promoter (P_*comK*_) during growth in the competence medium using a P_*comK*_*-luc* transcriptional fusion [88]. We observed an increasing expression from the P_*comK*_ promoter in WT cells up to the entry into stationary phase (Fig 3C). In the same time, P_*comK*_ activity was reduced about three-fold in Rho^+^ cells compared to WT (Fig 3C), although it remained higher than the basal expression level observed in the *spo0A* mutant **(**S4 Fig). Thus, we hypothesized that *comK* expression in Rho^+^ cells is insufficient to assure a threshold level of ComK required for competence induction [90].

In exponentially growing *B*. *subtilis* cells, transcription of *comK* is repressed by AbrB, Rok, and CodY [50,91,92]; it is activated by Spo0A~P, which also relieves the AbrB- and Rok-mediated repression thus opening a temporary “competence window” [88,93] (Fig 3A). Considering the low levels of *spo0A* expression, it was plausible that Spo0A-mediated de-repression of *comK* was inefficient in Rho^+^ cells. Thus, we attempted to increase expression of *comK* by inactivating its known repressors.

We first evaluated the significance of CodY-mediated regulation of *comK*, as repression activity of CodY does not depend on Spo0A~P [48,50]. In both WT and Rho^+^ cells, the *codY* mutation reduced the growth rate in the competence-inducing medium and stimulated P_*comK*_ activity less than 2-fold. As a result, the activity of P_*comK*_ in Rho^+^ *codY* cells remained below the WT level (S4 Fig). Not surprisingly, Rho^+^ *codY* mutant strain appeared non-transformable (Fig 3E). Next we showed that introduction of single *abrB* and *rok* mutations in WT cells increased activity of the P_*comK*_ promoter about 2- and 3-fold, respectively (S4 Fig), and simultaneous inactivation of both repressors synergistically stimulated *comK* expression (Fig 3D). Concordantly, we observed the increased transformation efficiencies of the mutants (Fig 3E). Inactivation of *abrB* or *rok* genes in Rho^+^ cells also led to the de-repression of P_*comK*_, close to or above its expression level in WT cells, respectively (S4 Fig), and the combination of both mutations led to a synergetic 5-fold increase of P_*comK*_ activity compared to WT cells (Fig 3D). However, despite the strong stimulation of *comK* expression, Rho^+^ cells mutated for *rok* and *abrB* remained non-transformable (Fig 3E). Therefore, we verified whether the de-repression of *comK* gene in Rho^+^ *abrB rok* mutant cells indeed resulted in activation of the competence genes. For this purpose, we used the P_*comGA*_-*gfp* transcriptional fusion widely applied as a single-cell reporter of the competence development within heterogeneous *B*. *subtilis* population [90, 94]. Fluorescence microscopy showed that in the competence medium, the proportion of Rho^+^ P_*comGA*_-*gfp abrB rok* cells expressing GFP was comparable to the WT P_*comGA*_-*gfp abrB rok* mutant and significantly higher than that of WT P_*comGA*_-*gfp* cells (~ 26% and 48% and 4% of the population, respectively) (Fig 3F and 3G).

These results indicate that Rho^+^ cells mutated for *abrB* and *rok* express ComK at a level sufficient for the induction of competence genes, but, nevertheless, stay transformation-negative. This suggests yet unknown roadblock(s) in the competence development and/or genetic transformation induced by an over-expressed Rho.

### Overview of transcriptome and proteome analyses of *B*. *subtilis* the Rho^+^ strain

To identify modifications of transcription presumably caused by *rho* over-expression, we performed RNAseq on Rho^+^, WT and Δ*rho* strains grown in LB medium. Two time points corresponding to the mid-exponential and early stationary phase were selected for this comparison (Materials and methods).

**Fig 4:**
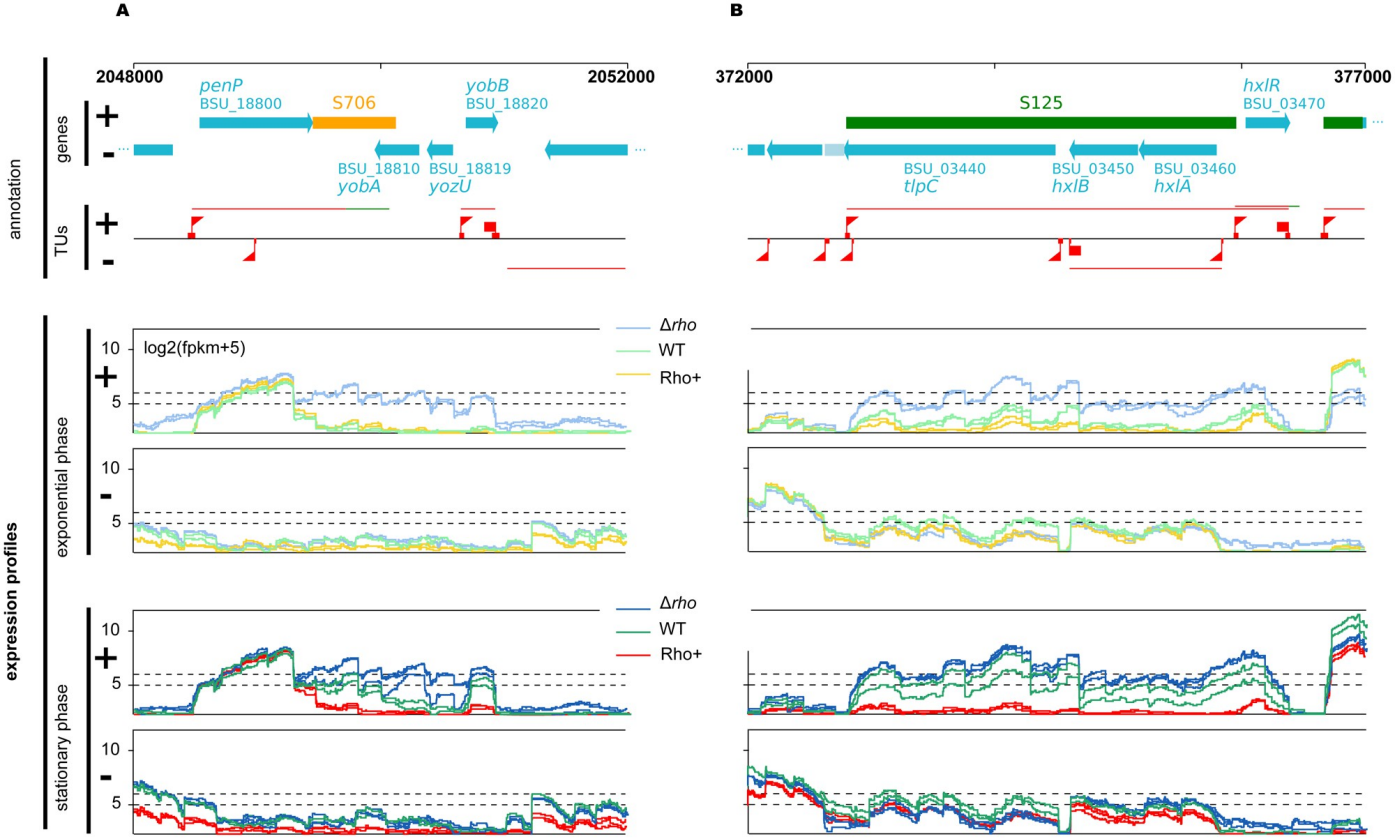
Rho-mediated transcriptional control. Examples of expression profiles of *B*. *subtilis* WT, Rho^+^ and Δ*rho* strains at two loci (**A** and **B**) as measured by RNAseq in exponential (middle panel) and stationary (bottom panel) growth phases for both strands of the genome (+ and -). The whole genome can be browsed at http://genoscapist.migale.inrae.fr/seb_rho. The top panel presents the structural organization of the region including the GenBank annotation, S-segments and transcription units (TUs) as determined from a compendium of WT expression profiles [15]. Triangle and square flags positioned on the TU lane (red) represent identified abrupt transcriptional up- and down-shifts often associated with promoter/terminator activity. The colors of expression profiles (middle and bottom panels) distinguish strains and growth phases: WT (light and dark green lines for exponential and stationary phase, respectively), Rho^+^ (orange and red) and Δ*rho* (light and dark blue). Inactivation of Rho in Δ*rho* mutant leads to the mRNA extension of the 3’UTR of *penP* gene (S706, antisense of *yobA-yozU*, sense of *yobB*) (**A**) and increases the expression of the asRNA (S125, antisense of *tlpC-hxlB-hxlA*, sense of *hxlR*) from its own promoter (**B**). Opposite effects are observed in Rho^+^. While Δ*rho* and Rho^+^ profiles are clearly distinguished in both conditions, the WT is intermediate with a position closer to Rho^+^ in exponential phase and to Δ*rho* in stationary phase (*i*.*e*. consistent with the decrease of Rho abundance upon transition to stationary phase in WT).

Exploration of the RNAseq data integrated two levels of analysis. At the gene level, we conducted differential expression (DE) analyses of sense and antisense strands of the native transcription regions (TRs) composed of 4,292 Genbank-annotated genes and 1,583 other TRs annotated as “Segments” (S-numbers) comprising ncRNAs and asRNAs [15]. The normalized gene expression (fpkm, fragments per kilobase of transcript per million fragments mapped) was compared between the samples. The results of these DE analyses are detailed in the next sections and S3 Table. In addition, we used Genoscapist [95] to set up a web site for interactive online exploration of strain- and condition-dependent quantitative expression profiles up to the single-nucleotide resolution; these profiles are illustrated in Fig 4 and can be accessed at http://genoscapist.migale.inrae.fr/seb_rho/.

To complement the RNAseq data, we assessed changes in a proteome composition associated with a steady high level of Rho during exponential growth and stationary phase (Materials and methods). In total, 1,465 unique proteins corresponding to 34% of the protein-coding *B*. *subtilis* genes were identified by mass spectrometry (S1 Table). To allow analysis of quantitative variations between WT and Rho^+^ proteomes, we used Protein Abundancy Index (PAI) values calculated from mass spectrometry data as described [96,97]: 175 and 193 proteins were found to be differently abundant (0.5 ≥PAI Rho^+^/WT≥ 2) in the exponential and stationary Rho^+^ cells, respectively, compared to WT. A number of proteins were found exclusively in Rho^+^ (28 and 73 for the exponential and stationary-phase cells, respectively), or in WT cells (76 and 92 for the exponential and stationary-phase cells, respectively) (S1 Table).

### At a high level, Rho suppresses antisense transcription genome-wide

**Fig 5:**
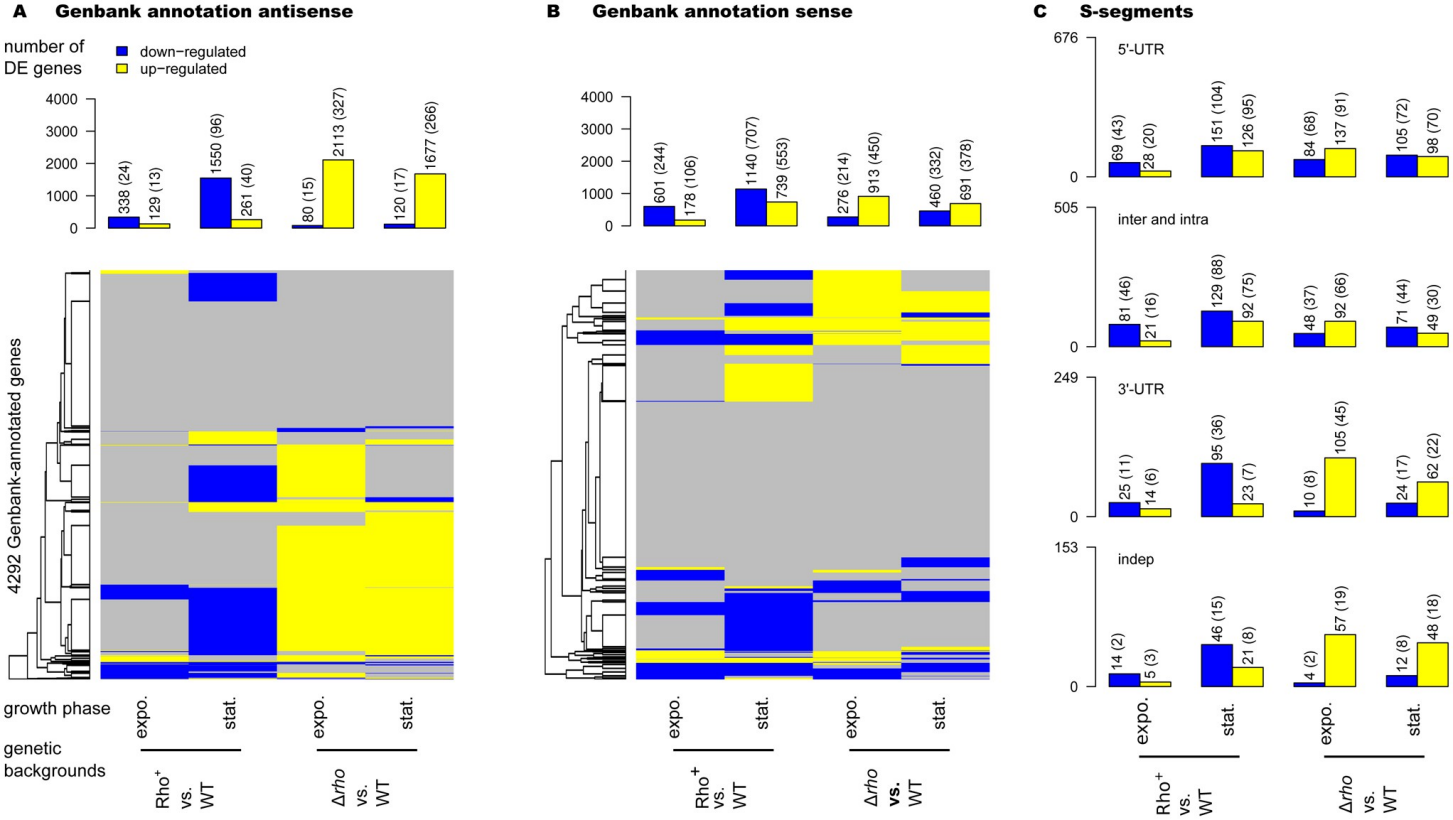
Graphical summary of differential sense and antisense expression (DE) in Rho^+^ and Δ*rho* across strands and growth phases. DE compared to WT (q-value≤0.05 and |log2FC|≥1) is shown for the antisense (**A**) and sense (**B**) strands of the 4,292 AL009126.3-annotated genes. *Up*: Barplot representation of the numbers of DE genes: numbers reported above each bar correspond to the total of DE genes and, in parentheses, to the subset exhibiting a minimal expression of log2(fpkm+5) ≥5 in one of the two compared genetic backgrounds. *Below*: Cluster heatmaps highlighting overlaps between these sets of DE genes: up-regulated genes in yellow, down-regulated genes in blue; all other genes, for which there is no statistical evidence for DE, are in gray. Left-side of each heatmap: average-link hierarchical clustering tree based on pairwise distance between genes (L1-norm after encoding down-regulation and up-regulation as -1 and 1, 0 otherwise). **(C**) Barplot representation of the numbers of DE S-segments, which were defined regarding to their locations as described in [15]: 5’- and 3’-UTR (for all 5’- and 3’-untranslated regions, respectively), inter and intra (for regions situated between genes), and indep (for independently transcribed segments). The up-regulated S-segments are in yellow, down-regulated are in blue. The number on the left side indicates the total number of S-segments of each defined category. The numbers reported above each bar correspond to the total of DE S-segments and, in parentheses, to the subset exhibiting a minimal expression of log2(fpkm+5) ≥5 in one of the two compared genetic backgrounds.

The RNAseq data revealed a global decrease of antisense transcription in Rho^+^ cells compared to WT (Fig 5A and S5 Fig and S3 Table), which mirrored the increase of antisense transcription already described in the Δ*rho* mutant [15] and confirmed by the present analysis (S3 Table). We found that transcription of antisense strand of 338 GenBank-annotated genes was down-regulated (log2 Rho^+^/WT ≤ -1; q-value ≤ 0.05) in the exponentially growing Rho^+^ cells, and this trend was even more pronounced in stationary phase where antisense transcription of 1,550 genes was down-regulated (S3 Table and S5 Fig). Of note, most of the antisense transcripts down-regulated in Rho^+^ cells (93% and 94% for exponential and stationary phase transcriptomes, respectively) are expressed in WT at levels that would be considered low for classical genes (log2(fpkm+5)≤5; Fig 5A). This is consistent with relatively low levels of antisense transcription in bacteria, part of which is often considered as transcriptional noise [98]. Therefore, only a minority of the genes, for which we report here a decreased antisense transcription in Rho^+^, were previously documented as being subject to antisense transcription in the WT *B*. *subtilis* strain [15].

Genoscapist-assisted examination of transcriptional profiles along the genome revealed modifications typical of an improved efficiency of Rho-specific and some intrinsic terminators. This enhanced termination prevents read-through transcription into adjacent, often convergent, genes (Fig 4A). Only 4% (14 out of 338) and 1.3% (20 out of 1550) of the down-regulated antisense transcripts (during exponential growth and stationary phase, respectively) were associated with decreased expression signal immediately after promoter (Fig 4B). Thus, the decreased transcription of the antisense strand in Rho^+^ cells is mainly a direct outcome of an enhanced termination activity of Rho at Rho-sensitive terminators.

### At a high level, Rho alters sense transcription mainly indirectly

Over-expression of *rho* caused considerable modifications of the sense-strand transcription, which, similarly to antisense transcription, were more pronounced in stationary phase (Fig 5B and S5 Fig and S3 Table). Considering the subsets of genes expressed above the cutoff of log2(fpkm+5) ≥5 in either of the two strains, 13% (553) of genes were found up-regulated and 16.5% (707) down-regulated in the stationary Rho^+^ compared to WT (1 ≤ log2 Rho^+^/WT≤ -1; q-value 0.05) (S3 Table).

Only 2% (15 out of 707) of the down-regulation events in stationary phase can be considered as resulting from the increased termination of transcription upstream or within the genes (*yxjL-M*, *ywhH*, *kbaA*, *kinB*, *ybbH-F-E-D-C*, *yqfT*, *yezA-yeeD*, *yvfH* and *ytpI*) preventing their transcription directly. The vast majority of the detected down-regulations were associated with a decrease in expression levels immediately after the promoters and, most likely, represent indirect regulatory effects of the improved activity of Rho, although direct termination of some promoter-proximal TECs by Rho [10,11] cannot be excluded either. The patterns of up- and down-regulated genes are not reciprocal in the Δ*rho* and Rho^+^ strains (Fig 5B). This suggests that the regulatory networks affected in Rho^+^ and Δ*rho* cells are not necessary the same.

Using the expression cutoff (log2(fpkm+5) ≥5), we also estimated the effect of Rho over-production on the expression of S-segments. The transcript levels were increased for 45 and decreased for 102 S-segments in the exponential Rho^+^ cells. More changes were observed (185 up- and 243 down-regulations) in stationary phase (Fig 5C and S3 Table). For 19.7% (42 out of 243) of S-segments down-regulated in the stationary Rho^+^ cells, expression levels are decreased due to an improved termination of transcription upstream of them, which may be a direct outcome of Rho activity (S4 Table). The 62% (26 out of 42) of S-segments directly down-regulated by Rho fall into the category of 3’ untranslated regions (3’UTR), which includes three types of 3′UTRs as defined in [15]: the proper 3′-UTR with clear termination signals; 3′-PT with partial termination, and 3′-NT without termination signals (S4 Table). None of the S-segments encoding ncRNAs with assigned physiological functions showed an altered expression level in Rho^+^ compared to WT.

To be able to retrace the propagation of the effects into regulatory cascades, we examined the correlation between DE and the known regulons and functional categories as defined in *Subti*Wiki database ([99]; http://subtiwiki.uni-goettingen.de). The complete list of statistically significant associations for up- and down-regulated genes in Rho^+^ and Δ*rho* (Fisher exact test p-value ≤ 1e-4) is presented in S5 Table. The strongest statistical associations between *B*. *subtilis* regulons and DE gene sets for the comparison Rho^+^ vs. WT in stationary phase were for AbrB, CodY, and the stringent response regulons (p-value ≤ 1e-12), forming the pathways governing the transition to stationary phase. Below we discuss the gene expression changes within these regulons in more details.

### Altered expression of the AbrB regulon in the stationary Rho^+^ cells

**Fig 6:**
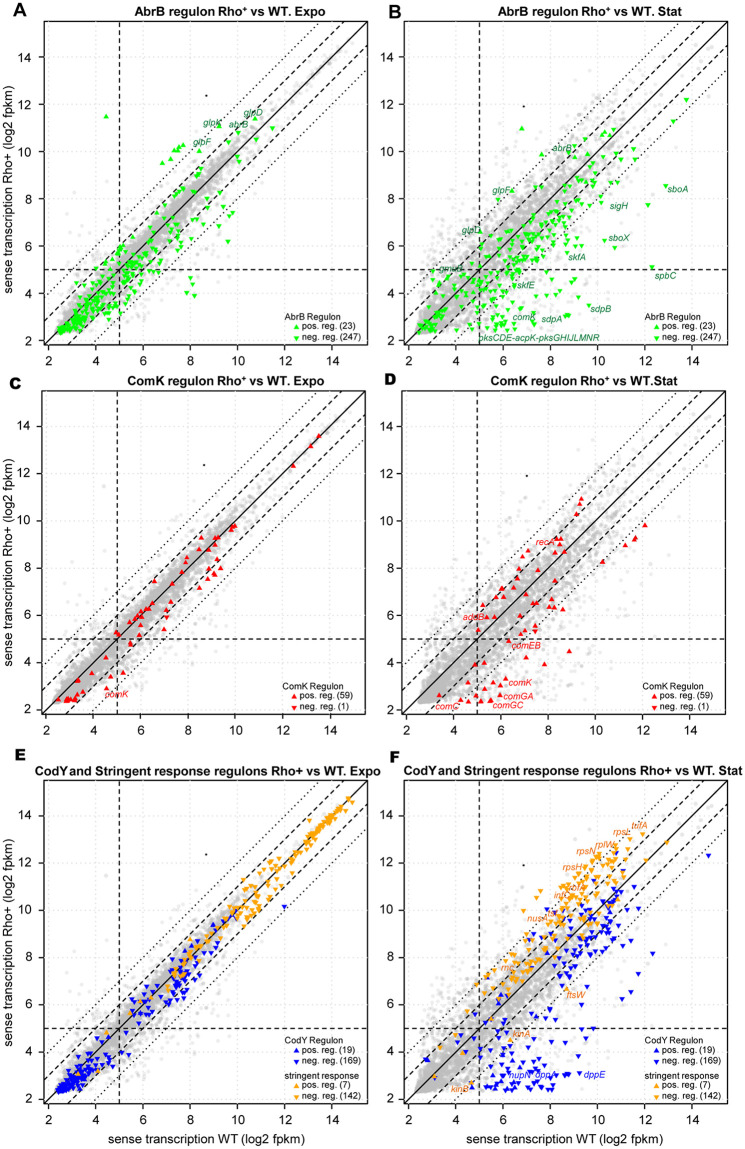
Differential expression of the AbrB, ComK, CodY and stringent response regulons in the Rho^+^ strain across exponential growth and stationary phase. Scatter plots display the expression values for the each gene from the AbrB regulon (A and B), ComK regulon (C and D), CodY and the stringent response regulon (E and F) by comparing *B*. *subtilis* Rho^+^ and WT strains under conditions of exponential growth (A, C, E) and stationary phase (B, D, F). Each symbol represents one of the 4,292 AL009126.3-annotated genes. Colored triangles indicate the genes from AbrB (green), ComK (red), CodY (blue) and the stringent response (orange) regulons, respectively; gray circles represent genes outside of the analyzed regulons. Expression levels of the individual genes in WT and Rho^+^ are represented, respectively, on x- and y-axes by the mean log2(fpkm+5) between biological replicates. Horizontal and vertical dashed lines correspond to the cut-off for minimal expression at log2(fpkm+5)≥5. The median diagonal solid line indicates unchanged gene expression levels, and genes close to it are not differently expressed. The diagonal dashed and dotted lines delineate DE changes of the |log2FC|≥1 and |log2FC|≥2, respectively. Within the analyzed regulons, the genes activated or repressed by cognate regulator are distinguished by the orientation of the vertex of corresponding triangles, and the number of genes is indicated in parentheses. For simplicity, only some of the genes mentioned in the text are indicated by their names. The complete DE changes within the regulons are summarized in S3 Table.

In Rho^+^ cells, the *abrB* transcript levels were 2-fold higher during exponential growth and in stationary phase compared to WT, which might be linked to a low activity of SpoA~P (Fig 6A and 6B and S3 Table). Accordingly, 36% and 62% of the negatively-controlled genes from the AbrB regulon were significantly down-regulated (log2 Rho^+^/WT ≤ -1; q-value ≤ 0.05) in the exponential- and stationary-phase Rho^+^ cells, respectively.

The largest difference between the strains was observed for the genes encoding secondary metabolites that mediate cellular growth and differentiation within the *B*. *subtilis* stationary population. For instance, the operons encoding antimicrobial compounds (*pksCDE-acpK-pksFGHIJLMNR*, *sunAT-bdbA-sunS-bdbB and sboA-sboX-albABCDEFG*), sporulation delay toxin SdpC (*sdpABC*) and the killing factor SkfA (*skfABCEFGH*) [100,101] were down-regulated up to 200-fold in stationary phase Rho^+^ cells compared to WT (e.g., log2 Rho^+^/WT = -7.4 for *pksF*, and -7.6 for *sdpC*; q-value = 0; S3 Table). Consistent with the transcriptome data, we detected a number of proteins encoded by AbrB-controlled genes (e.g., SunT, SunS, BdbA, PbpH, BacB) in the WT, but not in the Rho^+^ proteome (S1 Table).

Expression of the AbrB-controlled *sigH* gene encoding an alternative sigma factor regulating the genes involved in stationary phase adaptation [102] was increased in WT upon entering stationary phase, but was 4-fold lower in Rho^+^ (S3 Table). This effect propagated into the SigH regulon, since 35% of the SigH-dependent genes involved in stationary phase adaptation were down-regulated (log2 Rho^+^/WT ≤ -1; q-value ≤ 0.05) in Rho^+^ cells compared to WT (S5 Table).

In contrast, several genes known to be activated by AbrB and involved in the utilization of specific carbon sources (e.g., *glpD*, *glpFK*, and *gmuBACD*) [103,104] were upregulated in Rho^+^ compared to WT both during exponential growth and in stationary phase (Fig 6A and 6B and S3 and S5 Tables).

Overall, this analysis showed that the expression of the AbrB regulon genes, which enable the cells to adapt to suboptimal growth conditions, is significantly altered at a high Rho level.

### Suppression of the *ComK*-regulated genes in the Rho^+^ strain

In connection with AbrB, we examined the gene expression patterns of the ComK regulon. As expected from our analysis of the *comK* promoter activity (see above), the amount of the *comK* transcript was reduced in Rho^+^ cells compared to WT under exponential and stationary conditions ~ 4- and 15-fold, respectively (log2 Rho^+^/WT = -1.95 and -3.8, q-value = 0) (S3 Table and Fig 6C and 6D). Accordingly, 55% of genes from the functional category “Genetic competence” ([99]; http://subtiwiki.uni-goettingen.de) were down-regulated (log2 Rho^+^/WT ≤ -1; q-value ≤1.00e-04) in the stationary Rho^+^ cells compared to WT (Fig 6C and 6D and S3 and S5 Tables). The strongest reduction, more than 300-fold, was detected for the genes involved in binding and uptake of DNA [89]: the *comGA-GB-GC-GD-GE-GF-GG-spoIIIL*, *comEA-EB-EC* and *comFA-FB-FC* operons (e.g., log2 Rho^+^/WT ≤ -8.4 for *comGC*, q-value = 0; S3 Table). Meanwhile, transcript levels of some DNA recombination-repair genes belonging to ComK regulon, but known to be weakly dependent on ComK (e.g., *addBA*, *recA*) [105,106], were slightly increased in the stationary Rho^+^ cells compared to WT (Fig 6D and S3 Table).

We also analyzed the expression of 34 ncRNAs recently found to be strongly upregulated in the competent *B*. *subtilis* subpopulation [107]. As shown in S3 Table, 17 of them were under-represented in Rho^+^ compared to WT (log2 Rho+/WT ≤ -1.5; q-value ≤ 0.001).

In accordance with the previous data and the competence-proficient phenotype of the Δ*rho* mutant [15,26], no significant differences were detected for the expression patterns of the ComK-controlled genes in WT and Δ*rho* cells, with an exception of the *maf-radC* and *ykyB* genes up-regulated in the mutant during stationary phase (S6 Fig and S3 Table).

### Attenuated de-repression of the CodY regulon in the stationary Rho^+^ cells

The global transcription regulator CodY controls a number of genes essential for the transition from the exponential to stationary phase in accordance to the nutrient and energy cellular status [48, 52]. During exponential growth, all genes repressed by CodY are transcribed at a low level until the cells reach stationary phase when the activity of CodY starts to decline. In line with this, over 90% of the CodY-controlled genes were reliably activated in WT cells after the transition to stationary phase (S3 and S5 Tables). In contrast, the expression of more than 60% of the genes activated in the WT remained low in the stationary Rho^+^ cells (Fig 6F and S3 and S5 Tables).

Most of these genes encode proteins involved in amino acid metabolism and are controlled by one or more additional regulators (e.g., AbrB, TnrA or AhrC) responding to other intracellular and/or environmental signals. Others genes, like the *dppABCDE* operon encoding a dipeptide permease, or the *nupNOPQ* operon encoding the guanosine transporter, are under the sole control of CodY. Expression of these genes was strongly decreased in Rho^+^ cells compared to WT: from 25-fold for *dppA* to 190-fold for *dppE* (log2 Rho^+^/WT = − 7.7; q-value = 0; for *dppE*), and 34-fold for *nupN* gene (log2 Rho^+^/WT = − 5.1; q-value = 0) (S3 Table). In the WT proteome, 38 out of the 62 proteins encoded by genes negatively controlled by CodY were either exclusively found or over-represented in the stationary-phase proteome compared to the exponential phase; 22 of these proteins were not detected or under-represented in the stationary-phase Rho^+^ proteome (S1 Table).

Most of the genes down-regulated in the stationary-phase Rho^+^ cells fall into the clusters of genes, which were shown to be associated with the strongest CodY-binding sites and be repressed at an intermediate concentration of active CodY [108, 109]. All together, these results suggest that in the stationary-phase Rho^+^ cells, growth-dependent inactivation of CodY is inefficient or delayed compared to WT cells and that CodY partially retains the ability to control gene expression.

In Δ*rho* cells, de-repression of the CodY regulon in stationary phase was similar to WT, with the expression levels of 63% genes unchanged or differed from WT less than 2-fold (-1< log2 Δ*rho*/WT < +1; q-value < 0.05). At the same time, few genes known to be controlled by several transcription factors were found to be strongly up- (e.g. *ycgMNO*) or down-regulated (e.g. *frlBONMDP*) (S6 Fig and S3 and S5 Tables).

### Expression patterns of stringent response genes in WT and Rho^+^ cells are significantly different

Since CodY activity and the stringent response (SR) are tightly linked [53], inefficient de-repression of the CodY regulon in stationary Rho^+^ cells should be associated with altered expression of SR genes. This prompted us to compare the expression patterns of SR genes in the Rho^+^ and WT strains.

We found no significant difference in the expression of the SR genes in exponentially growing WT and Rho^+^ cells (Fig 6E and S3 and S5 Tables). As expected, expression levels of 87% (124 out of 142) of the genes negatively regulated by the SR were decreased more than 2-fold (log2 WT_stat_/WT_expo_ ≤ -1; q-value ≤ 1.00e-4) in WT cells entering stationary phase (S3 and S5 Tables). In accordance with the former transcriptional analysis of the SR [110], genes encoding the components of the translational apparatus, including ribosomal proteins (r-proteins) and translation factors were most strongly repressed. Of these genes, 91% showed at least a 4-fold decrease in their transcript levels, while 62% were down-regulated more than 10-fold (log2 WT_stat_/WT_expo_ ≤ -3.3; q-value ≤ 0.005; S3 Table). Conversely, the *kinA*, *kinB*, *ftsW* and *pycA* genes previously shown to be under positive stringent control [110–112] were up-regulated from 4- to 8-fold (2 ≤ log2 WT_stat_/WT_expo_ ≤ 3; q-value = 0.). These values fit well the changes in the expression of the stringent regulon genes detected earlier in WT cells [15].

The expression pattern of the stringent regulon was remarkably different in stationary phase Rho^+^ cells (Fig 6F and S3 and S5 Tables). In total, 60% of genes negatively regulated by the SR (86 out of 142) were expressed from 2- to 9-fold more efficiently in Rho^+^ cells than in WT (1 ≤ log2 Rho^+^/WT ≤ 3.2; q-value ≤ 4.00e-04). Almost all genes encoding r-proteins (e.g. *rpsH*, *rpsL*, *rpsN*) were up-regulated from 2- to 5-fold (1 ≤ log2 Rho^+^/WT ≤ 2.32; q-value = 0) in Rho^+^ cells compared to WT. The relative increase in transcript levels of genes encoding the translation factors (e.g., *tufA*, *tsf*, *fusA*, *infA*, *infB*, *infC*, *rbfA*) varied from 2- to 4-fold (1 ≤ log2 Rho^+^/WT ≤ 2; q-value = 0). A similar increase was observed for genes involved in RNA synthesis and degradation (e.g., *nusA*, *nusB* and *rnc*).

On the contrary, the *kinA*, *kinB* and *ftsW* genes were less efficiently expressed in Rho^+^ cells (Fig 6F and S3 Table). In Rho^+^, the expression level of the *kinB* gene was decreased 14-fold (log2 Rho^+^/WT = -3.8; q-value = 0) compared to WT. A low *kinB* expression in Rho^+^ cells was not exclusively due to the intragenic transcription termination described previously [26], as was also associated with a decreased expression level immediately after the *kinB* promoter. Notably, the transcription level of the *hpf* gene, which encodes a ribosome hibernation-promoting factor and is considered as a reporter for the activation of stringent response [113], was 2-fold lower in Rho^+^ compared to WT.

We noticed that some genes, which are known to be repressed during starvation in a Rel-independent manner [110], exhibit higher expression levels in the stationary Rho^+^ cells compared to WT, such as the genes from the purine and pyrimidine biosynthesis operons *purEKBCSQLFMNHD* (2.3 ≤ log2 Rho^+^/WT≤ 2.8, q-value = 0) and *pyrRPBCAAABKDFE* (2.4 ≤ log2 Rho+/WT≤ 4.2, q-value = 0), respectively. Accordingly, the amounts of PurM, PurQ, PyrB and PyrP proteins were increased in the stationary Rho^+^ proteome (S1 Table). These differences might reflect the altered levels of (p)ppGpp and GTP in Rho^+^ cells, as it has been shown that (p)ppGpp stimulates activity of PurR, the repressor of the *pur* operon [114], while GTP antagonizes the activity of PyrR, the repressor of the *pyr* operon [reviewed in 115].

Comparison of the WT and Δ*rho* transcriptomes did not reveal any global changes between the expression of the stringent regulon genes (S6 Fig and S3 and S5 Tables).

Overall, the present analysis showed significant differences between the transcription patterns of the SR genes in Rho^+^ and WT cells, which suggests that activation of the SR may be restrained in Rho^+^ cells. Following this hypothesis, we assessed characteristic phenotypes achieved through induction of the SR in *B*. *subtilis*.

### The Rho^+^ strain exhibits modified cell morphology and decreased stationary-phase survival

**Fig 7:**
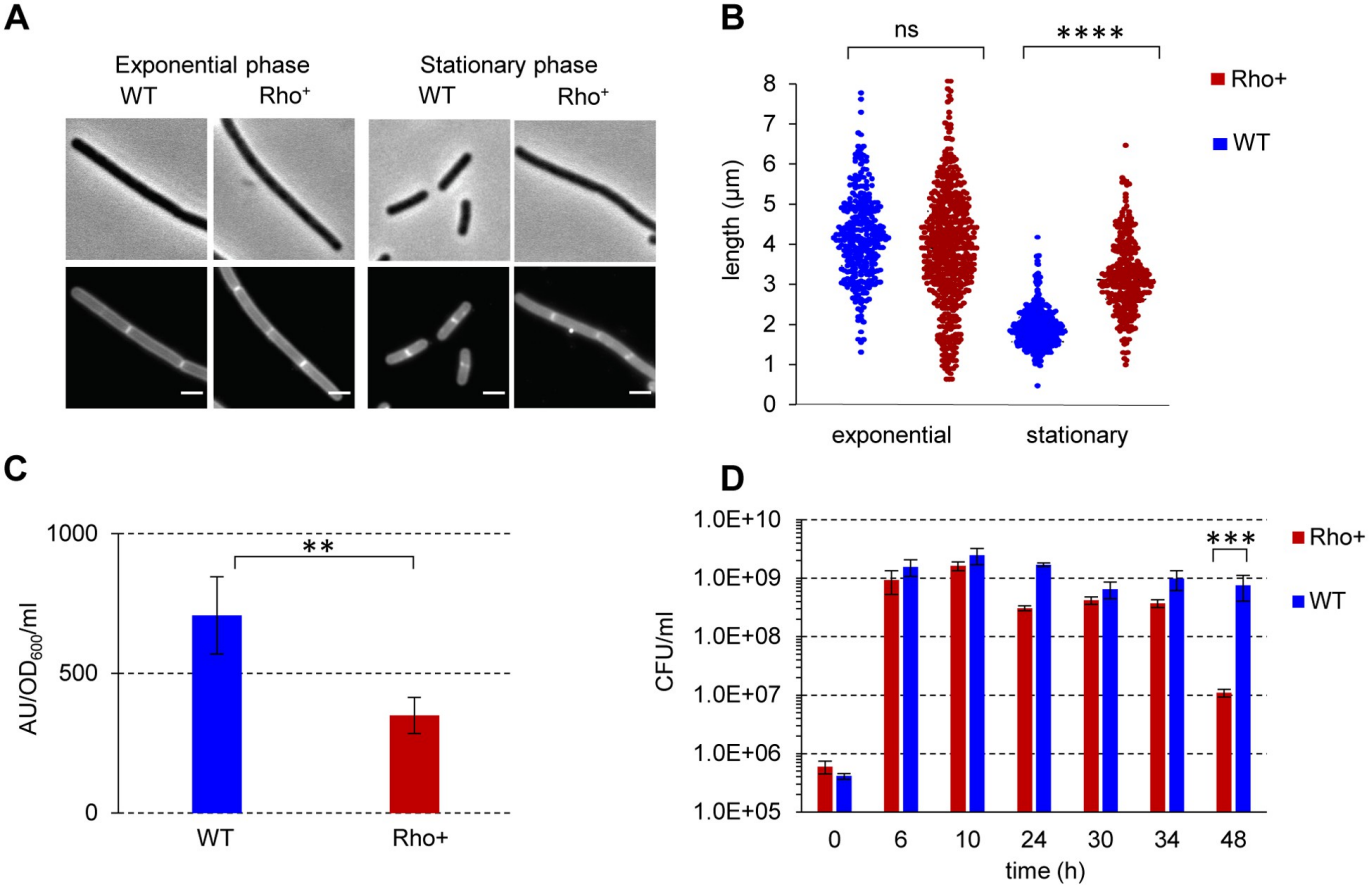
Morphology and long-term survival of *B*. *subtilis* Rho^+^ cells. Microscopy images (**A**) and cell length measurement (**B**) of *B*. *subtilis* WT and Rho^+^ under conditions of exponential growth and stationary phase. (A) Phase contrast (upper panel) and fluorescence (lower panel) images of WT and Rho^+^ cells stained with Nile Red at OD_600_ 0.2 and 1.8. Scale bars correspond to 2 μm. (B) The post-acquisition treatment of the images and determination of the mean cell lengths was as described in Materials and Methods. Statistical significance was estimated with a nested t-test, performed with Prism 9 (GraphPad Software, LLC). Note that the plot show the pooled values of the replicates. The displayed p-values are as follows: ****, p<0.0001; ns (non-significant), p>0.05 using a nested t-test. (**C**) Level of ppGpp under stationary phase. *B*. *subtilis* BSB1 WT and Rho^+^ cells were grown in defined SM medium supplemented with 0.5% (w/v) of casamino acids to stationary phase (OD_600_ 1.5). The ppGpp levels were assessed as described in Materials and Methods. Plotted are the mean values and SD from three independent experiments. The displayed p-value: **, p ≤ 0.01, using a two-tailed t-test. (**D**) Effect of Rho on long-term survival of *B*. *subtilis*. *B*. *subtilis* WT and Rho^+^ cells were grown in LB medium at 37°C with vigorous shaking during 48 hours. At the specified growth time, cells were plated on LB plates, and cell survival in cultures was assessed by the number of viable cells forming colonies (CFU) after 18 hours of incubation at 37°C. Plotted are the average values from three independent experiments each incorporating three biological replicas of each strain. The displayed p-value: ***, p ≤ 0.001 using a two-tailed t-test.

In bacteria, induction of (p)ppGpp synthesis and activation of the SR under starvation causes cell size reduction, so the (p)ppGpp-deficient cells are longer than WT cells, which correlates with an altered expression of genes involved in cell shape determination and biosynthesis of cell wall components [110,116–120]. This prompted us to compare the size of WT and Rho^+^ cells during exponential and stationary phases of growth in rich LB medium. We found no difference between the morphology of WT and Rho^+^ cells growing exponentially. However, while WT cells upon entry into stationary phase were effectively reduced in size and appeared as short rods (average cell length 1.9 ± 0.6 μm), Rho^+^ cells remained significantly longer (average cell length 3.2 ± 1.0 μm) (Fig 7A and 7B). In accordance with the observed decrease in WT cell size, the transcription levels of 53% of genes from the “Cell wall synthesis” *Subti*Wiki functional category [99] were decreased up to 10-fold in the stationary-phase WT (S3 Table). In Rho^+^ cells, most of these genes displayed higher expression levels. Notably, the transcript levels of the *murE-mraY-murD-spoVE-murGB* gene cluster involved in the biosynthesis of peptidoglycan precursors, the *cwlO*, *lytE* and *ftsX* genes encoding lytic enzymes critical for the cell elongation [121,122] were up to 4-fold higher (e.g., log2 Rho^+^/WT = 2.0; q-value = 0; for *cwlO*) in Rho^+^ compared to WT (S3 Table). To assess whether an abnormal size of the stationary-phase Rho^+^ cells correlates with a reduced level of (p)ppGpp alarmone, we compared the ppGpp pools in WT and Rho^+^ cells using high performance liquid chromatography (HPLC). Indeed, we found that Rho^+^ cells in stationary phase accumulated about two-fold less ppGpp compared to WT cells (Fig 7C).

Considering the crucial role of the SR in the adaptation and survival under starvation conditions [118,123–126], we next examined a long-term survival of Rho^+^ cells. The growth rate and viability of Rho^+^ cells during the exponential growth in LB medium were identical to those of WT (Figs 1 and 7D). However, while almost 50% of WT cells remained viable for at least 48 hours, the viability of the Rho^+^ strain decreased significantly during this time, as estimated by colony formation (Fig 7D). It is noteworthy that a decreased long-term survival of the Rho^+^ strain does not depend on its failure to sporulate, since LB medium does not support efficient sporulation, and at 48 hours, lesser than 0.5% of WT cells formed spores.

### The Rho^+^ strain exhibits phenotypic amino acid auxotrophy

**Fig 8:**
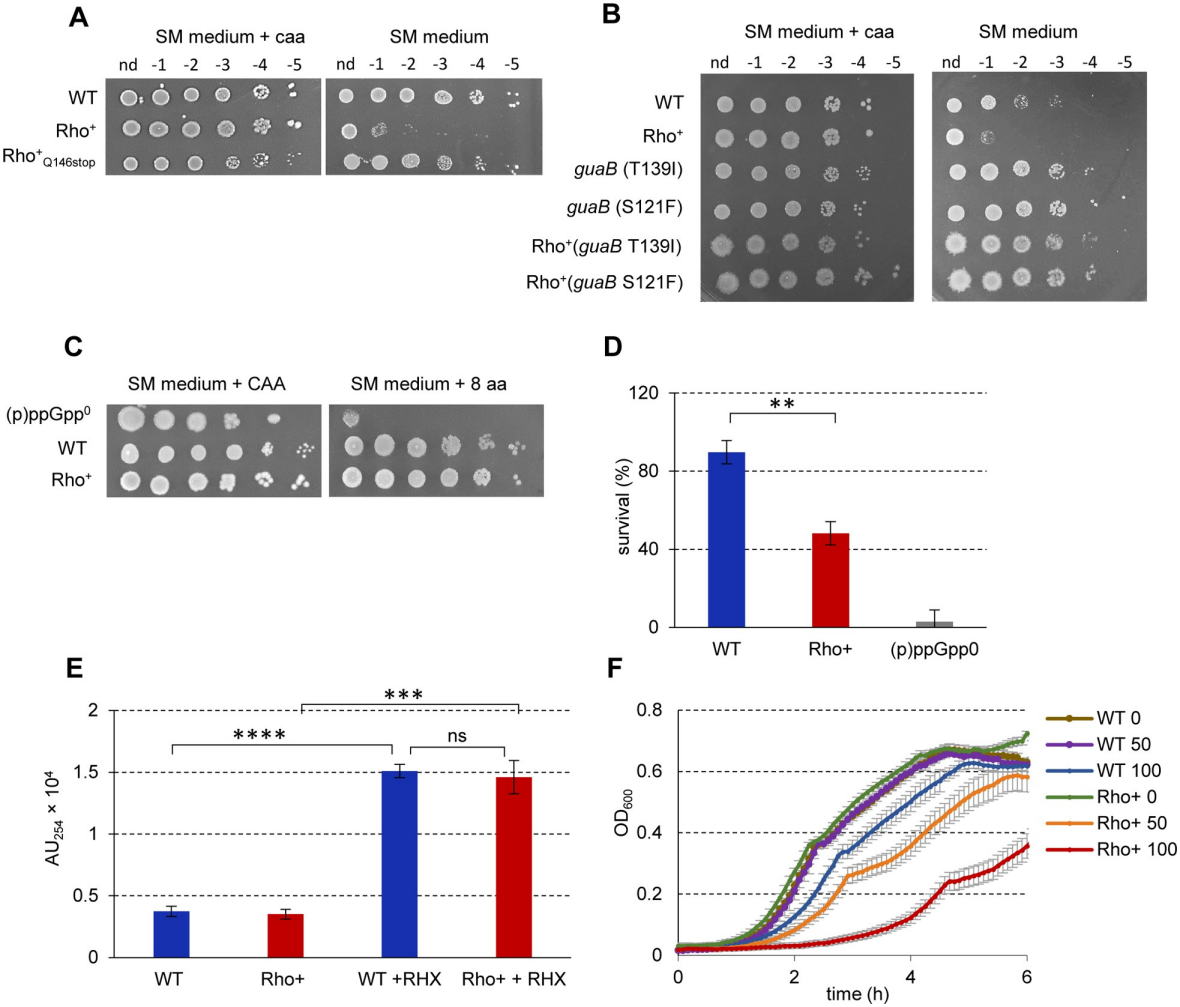
Adaptation of *B*. *subtilis* Rho^+^ cells to a sudden nutrient downshift. (**A**) Rho^+^ cells exhibit phenotypic amino acid auxotrophy. *B*. *subtilis* WT, Rho^+^ and Rho^+^_Q146Stop_ cells growing exponentially (OD_600_ 0.5) in liquid S7 medium containing 0.5% (w/v) of casamino acids (CAA) at 37°C were spotted in serial dilutions on SM agar plates supplemented or not with CAA (0.5%). Plates were incubated at 37°C during 18h before imagining. (**B**) Decreasing of GTP levels rescues the auxotrophic phenotype of Rho^+^ cells. *B*. *subtilis* WT, Rho^+^ and their respective derivative strains carrying *guaB*S121F and *guaB*T139I mutations were cultivated in liquid S7 medium containing 0.5% (w/v) of CAA and tested for the ability to grow in the absence of CAA as in (A). (**C**) Unlike (p)ppGpp^0^ cells, the Rho^+^ strain resists to mild nutrient limitations. Isogenic WT, (p)ppGpp^0^ and Rho^+^ strains were grown in liquid defined SM medium containing 0.5% (w/v) of CAA and spotted in serial dilutions on SM agar plates supplemented either with 0.5% (w/v) of CAA or with 0.05mg/ml of each of the eight amino acids (valine, isoleucine, leucine, threonine, histidine, arginine, tryptophan and methionine). Plates were incubated at 37°C during 18h before imaging. In (A, B and C), the experiments were reproduced at least three times and the representative results are shown. (**D**, **E** and **F**) Rho^+^ cells exhibit altered resistance to arginine hydroxamate (RHX). (**D**) Isogenic *B*. *subtilis* WT, (p)ppGpp^0^ and Rho^+^ strains were grown to the middle exponential phase (OD_600_ 0.5), treated with 500 μg/ml of RHX for 40 min and plated on LB agar plates. Plates were incubated for 18 h at 37°C before counting viable cells that formed colonies. Strain survival upon sudden amino acid starvation induced by RHX was estimated as the percentage of viable cells after and before the treatment. Plotted are the average values and SD from three independent experiments incorporating three biological replicas of each strain. Statistical significance was estimated with a two-tailed t-test. The displayed p-value: **, p ≤ 0.005. (**E**) Increase of ppGpp level following sudden amino acid starvation. *B*. *subtilis* WT and Rho^+^ cells were grown in SM medium supplemented with 0.5% (w/v) of CAA to the middle exponential phase (OD_600_ 0.5) and treated or not with 500 μg/ml of RHX. Cells were harvested 20 min after addition of RHX, and ppGpp levels were assessed as described (Materials and methods). Plotted are the average values and SD from three independent experiments. The displayed p-values are as follows: ****, p ≤ 0.0001; ***, p ≤ 0.001; ns (non-significant), p>0.05 using two-tailed t-test. (**F**) Growth defect of the Rho^+^ strain in the presence of RHX. *B*. *subtilis* WT and Rho^+^ strains were cultivated in LB medium without or with RHX added at concentrations 50 and 100μg/ml in a 96-well microplate. Growth of the cultures was monitored by OD_600_ measurement at the five-minute intervals using a microplate reader. Plotted curves are the average OD reads of two independent cultures of each strain grown in triplicates at each condition. The analysis was performed three times; the results of a representative experiment are presented.

*B*. *subtilis* mutants deficient in (p)ppGpp production ((p)ppGpp^0^) are characterized by phenotypic auxotrophy for amino acids, in particular, BCAA, threonine, histidine, arginine, tryptophan and methionine, provoked by a deregulation of GTP homeostasis [60,65,127]. Therefore, we tested the ability of Rho^+^ cells to form colonies on minimal SM medium either in the presence or absence of amino acids (Materials and methods). Both WT and the Rho^+^ strains grew equally well on SM medium supplemented either with casamino acids (CAA) or with eight amino acids listed above (Fig 8A; data shown for SM medium supplemented or not with CAA). However, omission of CAA had a strong inhibitory effect on the colony-forming ability of the Rho^+^ strain, contrary to WT or the Rho^+^_Q146Stop_ mutant (Fig 8A).

It was previously shown that lowering the intracellular level of GTP restores the viability of *B*. *subtilis* (p)ppGpp^0^ cells in minimal medium without CAA [60,65,127]. To analyze whether this was also true for cells over-expressing Rho, we decided to decrease the level of GTP in the Rho^+^ strain by mutating *guaB*, the essential gene of the GTP biosynthesis pathway. This was performed by introducing, in WT and the Rho^+^ strains, the partial loss-of-function point mutations *guaB* T139I and *guaB* S121F, which were previously isolated as spontaneous suppressors of the poly-auxotrophy of *B*. *subtilis* (p)ppGpp^0^ mutant [60]. Both T139I and S121F *guaB* mutations rescued the auxotrophic phenotype of the Rho^+^ strain (Fig 8B), reinforcing a potential link between a high Rho content and the shortage of (p)ppGpp in Rho^+^ cells.

However, Rho^+^ cells did not exhibit some other phenotypes characteristic of the absence of (p)ppGpp. Indeed, while (p)ppGpp^0^ cells adapt poorly to a sudden nutrient downshift and cannot survive the transition from amino acid-replete medium to amino acid-limited medium [65], Rho^+^ cells propagated in liquid defined SM medium containing CAA formed colonies on a solid SM medium supplemented with only eight amino acids (Fig 8C).

Rapid death upon treatment with a nonfunctional amino acid analog arginine hydroxamate (RHX), an inhibitor of arginyl-tRNA synthesis and a powerful activator of the SR, is other distinctive feature of (p)ppGpp^0^ cells [60,127]. However, following the published protocol of pulse treatment of cells with RHX [65], which led to the death of (p)ppGpp^0^ cells, we observed a rather minor ~2 fold effect of RHX on the viability of Rho^+^ cells compared to WT cells (Fig 8D). Moreover, in response to pulse RHX treatment, the ppGpp pool increased similarly in the exponentially growing WT and Rho^+^ cells (Fig 8E). Nevertheless, Rho^+^ cells appeared highly sensitive to constant exposure to RHX during growth in rich LB medium or in defined SM medium supplemented with CAA (Fig 8F).

Taken together, these findings converge to the hypothesis that Rho^+^ cells have a reduced capacity to synthesize (p)ppGpp and to induce the SR under some stressful conditions.

### The Rho^+^ strain is sensitive to fatty acid starvation and heat stress

It has been shown that (p)ppGpp deficiency caused exclusively by inhibition of the synthetic activity of the bifunctional synthetase-hydrolase Rel, results in a high sensitivity to fatty acid starvation and heat stress [119,128].

**Fig 9:**
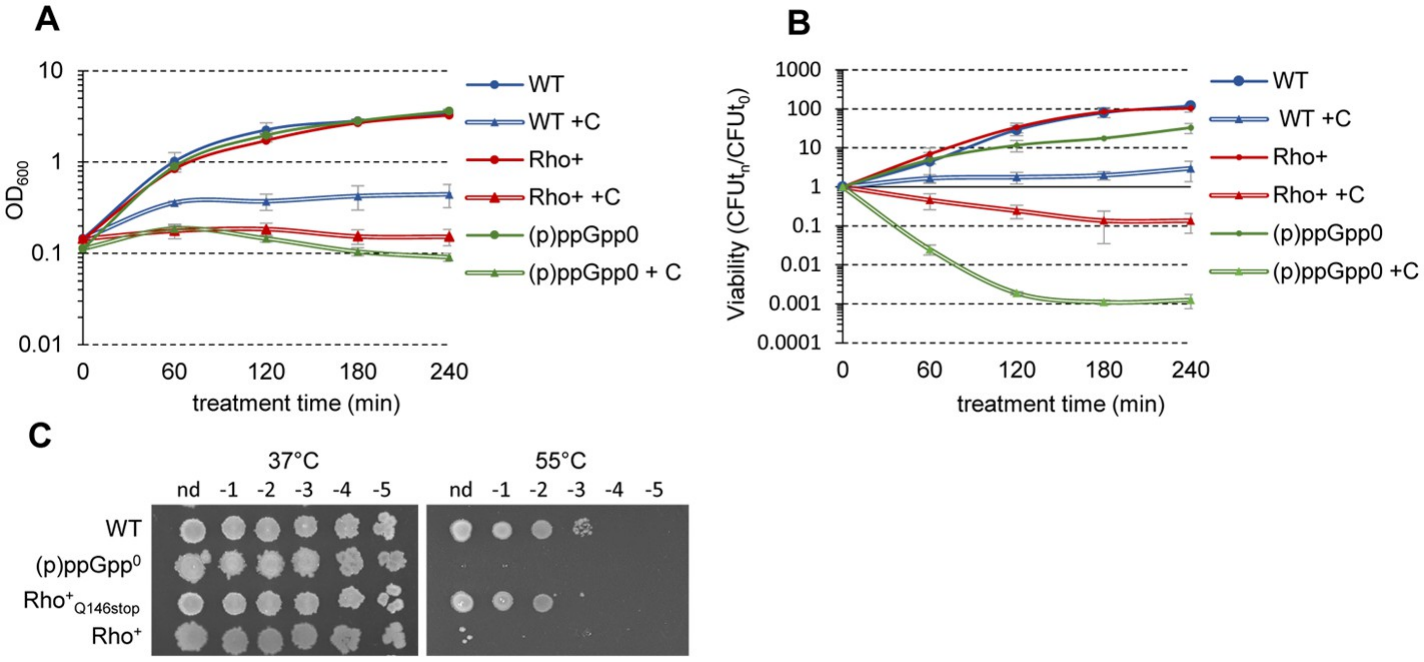
Sensitivity of *B*. *subtilis* Rho^+^ to fatty acid starvation and heat stress. Growth curves (**A**) and viability (**B**) of *B*. *subtilis* WT (blue lines), its isogenic (p)ppGpp^0^ (green lines) and Rho^+^ (red lines) strains non-treated (solid lines; circles) or starved for fatty acids by treatment with cerulenin (+ C; double lines; triangles). Cells were grown in LB at 37°C to OD_600_ 0.1, and cerulenin was added to the halves of cultures to final concentration 10μg/ml. Cell survival was estimated by plating bacterial cultures at the indicated time on LB agar and counting of CFUs after 18 h of incubation at 37°C. Each data point in B is the mean of at least three counts. (**C**) Colony formation of *B*. *subtilis* WT and its isogenic (p)ppGpp^0^, Rho^+^ and Rho^+^_Q146Stop_ (Sup 1) strains at 37°C and 55°C. Cell cultures growing exponentially in LB medium at 37°C were spotted in serial dilutions (from 0 to 10^−5^) on LB agar plates and incubated at 37°C or at 55°C for 18 h before imagining.

We assessed the ability of Rho^+^ cells to adapt to fatty acid starvation using cerulenin, an inhibitor of the fatty acid synthesis enzyme FabF [129]. As reported previously [119], treatment with cerulenin did not affect viability of WT cells but appeared highly toxic for the (p)ppGpp^0^ strain. Addition of the drug had a less pronounced but significant inhibitory effect on Rho^+^ cells resulting in an efficient growth arrest and loss of viability as assessed by colony formation (Fig 9A and 9B).

Next, we examined the growth capacity of WT and Rho^+^ cells at 55°C, the temperature shown to be non-permissive for the synthetase-deficient *rel* and (p)ppGpp^0^ mutants [128]. While the colony-forming capacity of WT strain was not affected at 55°C, the Rho^+^ strain did not form colonies at this temperature (Fig 9C). Furthermore, the thermo-sensitive phenotype of the Rho^+^ strain allowed us to isolate the mutants able to grow at 55°C. The subsequent analysis of several thermo-resistant Rho^+^ clones revealed mutations of the ectopic *rho* expression unit (S2 Table) and confirmed that a high level of Rho underlies the heat sensitivity of Rho^+^ cells. Notably, lowering the cellular GTP level by the partial loss-of-function mutations *guaB* T139I and *guaB* S121F did not rescue the thermo-sensitivity of the Rho^+^ strain (S7 Fig).

Thus, we hypothesize that the cellular levels of (p)ppGpp produced by the major alarmone synthetase Rel in Rho^+^ cells under fatty acid starvation and heat shock are insufficient to confer stress resistance.

## Discussion

*B*. *subtilis* adapts to adverse environmental conditions by various strategies ranging from the adjustment of metabolic processes via the activation of the stringent response (SR) to sporulation as an ultimate survival option.

Previously, using a Δ*rho* mutant we showed that the transcription termination factor Rho negatively affects the activity of Spo0A, the master regulator of *B*. *subtilis* differentiation, by controlling its activation through the phosphorelay. We also identified several asRNAs, which result from the read-through at Rho-controlled terminators and contribute to coordinated regulation of cell differentiation independently from Spo0A [26].

**Fig 10:**
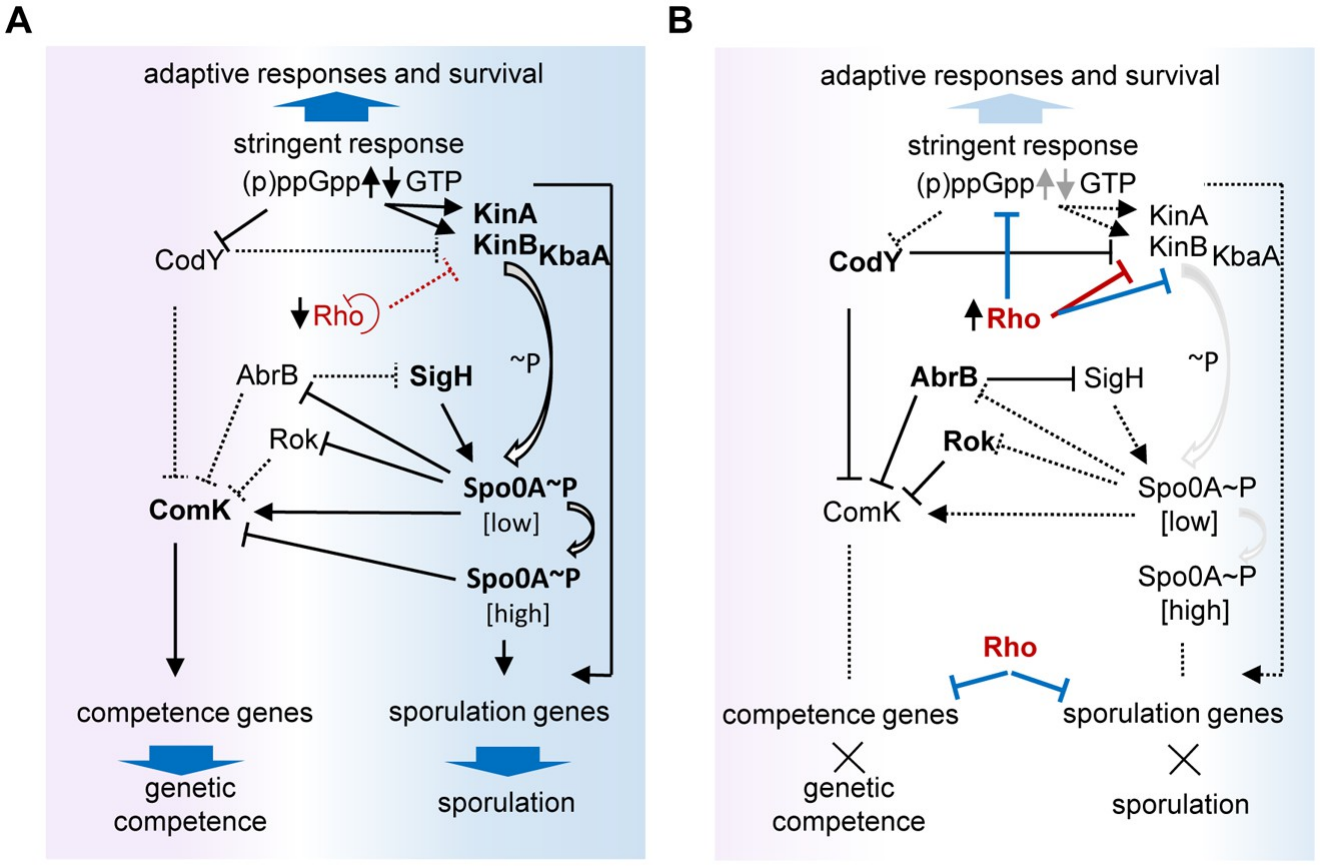
Tentative model integrating the termination factor Rho into the regulatory networks controlling the adaptation to stationary phase and the programs of genetic competence and sporulation in *B*. *subtilis*. For simplicity, only the key elements relevant to this study are shown, and the discussion of the regulatory activities of CodY and AbrB is limited to the *comK* gene. The arrowhead lines and bar-headed lines indicate positive and negative regulation, respectively. Solid and dashed headed lines indicate strong and weakened regulation, respectively, and the dashed lines without extremities stand for the loss of positive regulation. Red and blue bar-headed lines depict the Rho-mediated negative regulatory events identified previously (red) and in this study (blue). (**A**) In *B*. *subtilis* cells grown under starvation conditions, the increased (p)ppGpp synthesis linked to the activation of stringent response leads to lowering of GTP levels, thus decreasing the activity of CodY and stimulating the expression of the *kinA* and *kinB* genes, which are under positive stringent control [112]. The major sensor protein kinases KinA and KinB initiate transfer of the phosphoryl groups to Spo0A. KbaA protein stimulates KinB activity. The gradual increase in Spo0A∼P activates expression of *comK* directly and indirectly by relieving the Rok- and AbrB-mediated repression of *comK*. By repressing AbrB, Spo0A~P activates the expression of *sigH*, thus creating a positive feed-back loop of autoregulation [37,79]. When concentration of Spo0A~P reaches a high threshold level, it represses *comK* and activates expression of early sporulation genes. Synthesis of (p)ppGpp is necessary throughout the entire sporulation pathway [126]. In starving cells, Rho levels decrease due to the negative autoregulation [72] and some unknown regulatory circuits. Lowering of the Rho levels decreases the efficiency of the Rho-controlled transcription terminators thus activating, directly and indirectly, transcription of a set of genes and ncRNAs involved in adaptation to stationary phase. In particular, the lowering of Rho weakens the activity of the *kinB* intragenic Rho-dependent transcription terminator; as a result, the intensified read-through at the terminator stimulates synthesis of the full-length *kinB* transcript and consequently the activity of Spo0A~P [26]. (**B**) Counteracting the natural decrease of Rho levels by *rho* over-expression alters the normal progression to stationary phase as depicted. Stabilization of the intracellular levels of Rho in the stationary Rho^+^ cells improves the Rho-dependent transcription termination and consequently alters the expression of the Rho-controlled genes and ncRNAs. A high Rho inhibits read-through at the intragenic transcription terminators of *kinB* and the newly identified Rho target *kbaA* gene thus decreasing the full-gene transcription of *kinB* and *kbaA*. A decreased flux of the phosphate alters the Spo0A~P-mediated inhibition of AbrB and thus disrupts its positive autoregulatory loop. As a result, Spo0A~P does not reach the threshold levels necessary neither for the de-repression of *comk*, nor for the efficient activation of early sporulation genes. The increase in Rho levels reinforces a still unknown mechanism by which Rho modulates the synthesis of (p)ppGpp and regulates the activation of stringent response. Altering the balance between p(p)ppGpp and GTP levels leads to an increased repression of *comK* by CodY and a weak activation of the positively-regulated stringent genes *kinA* and *kinB*, which additionally contributes to decreasing Spo0A~P activity. In addition, the analysis of the Rho^+^ strain pinpoints the existence of yet non-identified targets of Rho-mediated regulation among the competence and sporulation genes. Overall, the increased control of Rho over its multiple targets provokes the loss of competence and sporulation in Rho^+^ cells.

Here, we provide evidence that Rho is involved in the complex regulatory network that governs the transition to stationary phase and integrates the global transcription factors Spo0A, AbrB, CodY, and (p)ppGpp, an alarmone triggering the SR. Our results demonstrate that maintaining Rho expression at a stable elevated level modifies genome-wide gene expression and causes pleiotropic effects on cellular physiology and cell-fate decision-making to such an extent that it blocks the competence development and sporulation. A simplified scheme of this regulation is presented in Fig 10. The decrease in Rho levels occurring during the transition to stationary phase [15,26] therefore appears to be important for the functionality of these regulatory networks.

The RNAseq analysis revealed that Rho over-production led to a genome-wide increase of termination efficiency on the Rho-sensitive terminators, thus reducing read-through transcription, which is antisense for convergent genes. The Rho-dependent termination appears therefore to be a heterogeneous process modulated by the amount of Rho and structural features of its targets, as it was previously shown for the global regulators of transcription Spo0A and CodY [80,93,94,108,109].

Global inhibition of read-through transcription in Rho^+^ cells emphasizes the role of Rho in the suppression of pervasive transcription regardless of whether the resulting 3’UTR transcripts (both sense and antisense) are strongly expressed or represent a transcriptional noise. However, the consequences of this genome-wide effect are difficult to evaluate, as regulatory mechanisms employed by 3’UTRs transcripts and in particular, by asRNAs, are diverse and only few of them have been functionally characterized in *B*. *subtilis* [130–132]. In light of the emerging functional significance of low-level pervasive transcription in bacteria [29,133], its suppression in Rho^+^ cells can also potentially affect cellular physiology. At the same time, the reliably identified direct effects of Rho over-production on the sense transcription are rare, as down-regulation events due to the improved transcription termination affect only 15 protein-coding genes and 42 S-segments (expressed above the threshold of log2(fpkm+5)≥5 in WT), while hundreds occur at or closely behind the promoters and are probably indirect. Nevertheless, considering the established role of the *Escherichia coli* Rho in transcriptional pausing via direct association with elongating RNA polymerase [10,11], a part of the Rho-mediated down-regulation of the antisense and sense strands might result from an improved transcription termination close to promoters. More analysis is needed to understand the promoter-proximal down-regulation events induced by Rho in *B*. *subtilis*.

It is not clear yet how these primary events can contribute to the altering of stationary phase in Rho^+^ cells. An obvious exception is a direct effect of Rho on a premature transcription termination of the *kinB* and *kbaA* genes encoding sensor kinase and its positive effector, which is consistent with the low activity of Spo0A~P in Rho^+^ cells (Fig 10). However, while emphasizing the importance of Spo0A~P for cellular adaptive responses, our results show that its low level is not a sole cause of stationary phase reprogramming and the competence- and sporulation-negative phenotypes of Rho^+^ cells. Indeed, although the over-production of KinA kinase in Rho^+^ cells significantly improved their entry into the sporulation program, plausibly by increasing Spo0A~P levels, it did not rescue the formation of heat-resistant spores. Likewise, induction of *comK* expression in the Rho^+^ *abrB rok* mutant above the WT threshold was sufficient for activation of the essential competence genes, but it did not restore the impaired transformability of the Rho^+^ strain. Thus, expressing Rho at a high level generates additional roadblocks in these differentiation pathways. Given the large and ever-growing number of genes and ncRNAs involved in the development of genetic competence and spore formation [105–107,131,134–138], we suggest that Rho negatively regulates some of them. We expect that these issues will be resolved in future studies.

The role of Spo0A~P goes beyond the regulation of genetic competence and sporulation (Fig 10A). Through the control of AbrB, Spo0A~P mediates the de-repression of genes important for adaptation to stationary phase including *sigH* [39,104]. Thus, the repression of the AbrB and SigH regulons is consistent with low Spo0A~P in Rho^+^ (Fig 10B).

The activity of CodY, the second transition-state regulator, is independent from Spo0A~P and controlled by GTP, whose levels decrease upon entering stationary phase alongside the increased synthesis of (p)ppGpp [54,59–61,70] (Fig 10A). The RNAseq analysis unveils that over-production of Rho coincides with the prolonged repression of the CodY regulon and the weakening of gene expression changes characteristic of the SR. Consistently, we detected ppGpp in the stationary Rho^+^ cells at a half of the WT level and showed that the Rho^+^ strain exhibits several hallmarks of (p)ppGpp-deficiency [65,119,128].

The (p)ppGpp is involved in the control of genetic competence by lowering GTP levels, thereby modulating the CodY activity [50,51]. A drop in the GTP levels is a well-known sporulation trigger [59,70,138–140] due to the activation of the *kinA* and *kinB* genes, which are under positive stringent control depending on adenine as the transcription initiation nucleotide [111,112] (Fig 10A). In addition, *kinB* is under the direct negative control of CodY [49]. Thus, both *rel* and (p)ppGpp^0^ mutants are characterized by a delay in *spo0A* expression [71,141]. Moreover, synthesis of (p)ppGpp is crucial throughout the entire pathway of sporulation. This follows from the study of a specific inhibitor of the Rel-mediated (p)ppGpp synthesis Relacin, which inhibited formation of spores regardless the time at which it was added to cells committed to sporulation [126]. Therefore, attenuation of (p)ppGpp synthesis might provide additional level of negative regulation of competence development and sporulation in Rho^+^ cells (Fig 10B).

In *B*. *subtilis* cells, accumulation of (p)ppGpp is determined by joint activities of the alarmone synthetases and hydrolases [113,142–144]. Whereas the expression of both small (p)ppGpp synthetases RelP and RelQ is mainly controlled at the transcriptional level, the bifunctional synthetase-hydrolase enzyme Rel is under allosteric regulation [57,58,145]. According to the RNAseq analysis, the expression levels of the *rel*, *relP* and *relQ* genes, and (p)ppGpp hydrolase gene *nahA* were similar in Rho^+^ and WT cells (S3 Table). Since synthesis of (p)ppGpp by Rel is essential for thermo-resistance and survival under fatty acid starvation [119,128], we suggest that partially relaxed phenotype of the Rho^+^ strain may be associated with insufficient accumulation of (p)ppGpp mediated by this bi-functional enzyme.

It is known that the synthetase and/or hydrolase activities of some (p)ppGpp enzymes are modulated by direct protein-protein interactions. In *E*. *coli*, YtfK and the acyl carrier proteins trigger synthetic activity of synthetase-hydrolase SpoT, while NirD protein inhibits monofunctional synthetase RelA [146–148]. In *B*. *subtilis*, synthesis of (p)ppGpp by Rel is stimulated through its interaction with cyclic-di-AMP-binding protein DarB [149], while the late competence protein ComGA specifically inhibits the hydrolase activity of Rel leading to a temporary increase of the (p)ppGpp pool [150]. However, a role for these proteins in Rel-mediated metabolism of (p)ppGpp in Rho^+^ cells seems unlikely. First, the levels of c-di-AMP, which inhibits DarB-Rel interaction [149], are likely similar in WT and Rho^+^ cells under the applied conditions, as suggested by the unchanged expression of the *ktrA* gene controlled by a c-di-AMP-dependent riboswitch [151] (S3 Table). Second, although ComGA is not expressed in Rho^+^ cells due to the repression of ComK, which would stimulate (p)ppGpp hydrolysis, the previous transcription analysis of the *comK* mutant revealed no changes in gene expression indicative of an altered Rel activity [107]. In addition, unlike Rho^+^ cells, the *comK* and *comGA* mutants are heat-resistant (S7 Fig).

Albeit the precise molecular mechanism by which Rho delays the accumulation of (p)ppGpp and weakens the stringent response awaits further investigation, the unexpected link between Rho and (p)ppGpp metabolism should be of particular interest given the importance of this secondary messenger for virulence, antibiotic resistance and persistence in bacteria [126,152–155]. Our results may also be relevant to other bacterial species for which Rho has been shown to be involved in the control of various stationary-phase associated phenomena (cell fate decisions, virulence, antibiotic sensitivity, stress resistance and survival under restrictive conditions) [26,33–35,156–163].

In conclusion, our study highlights the importance of factor-dependent and, in particular, Rho-mediated transcription termination in regulation of both coding and non-coding transcriptomes, as well as its role in the control of complex physiological processes of bacteria.

## Materials and methods

### Bacterial strains and growth conditions

*B*. *subtilis* strains used in the work are derivatives of 168 *trp*^+^ BSB1 [15] and listed in S6 Table. Cells were routinely grown in Luria-Bertani liquid or solidified (1.5% agar; Difco) medium at 37°C. Where indicated, S7 defined synthetic medium [164] containing 50 mM 3-(*N*-morpholino)propanesulfonic acid (MOPS) and supplemented with 0.1% (wt/vol) glutamate, 0.5% (wt/vol) glucose, 0.5% (wt/vol) casamino acids (Difco), and 0.01% (wt/vol) tryptophan was used. To determine the level of ppGpp, cells were grown in the liquid SM medium (10.8 g l^−1^ of K_2_HPO_4_, 6 g l^−1^ of KH_2_PO_4_, 1 g l^−1^ of C_6_H_5_Na_3_O_7_.2H_2_O, 0.2 g l^-1^ of MgSO_4_.7H2O, and 2 g l^−1^ of K_2_SO_4_) supplemented with 0.5% glucose, 0.5% casamino acids (Difco), 0.01% L-tryptophan, 0.016% L-glutamine, 0.1 mM of FeCl_3_ citrate, 0.1 mM of CaCl_2_, 1 mM of MgSO_4_ and trace elements (0.001 g l^−1^ of MnCl_2_ 4H_2_O, 0.0017 g l^−1^ of ZnCl_2_, 0.00043 g l^−1^ of CuCl_2_· 2H_2_O, 0.0006 g l^−1^ of CoCl_2_ 6H_2_O and 0.0006 g l^−1^ of Na_2_MoO_4_· 2H_2_O). For amino acids auxotrophy tests, cells were plated on the solidified (1.5% agar; Difco) supplemented SM medium, in which L-glutamine was substituted by 0.1% (wt/vol) glutamate and casamino acids (Difco) were added at 0.5% or 0.004% (wt/vol).

Standard protocols were used for transformation of *E*. *coli* and *B*. *subtilis* competent cells [164]. Sporulation was induced either by the nutrient exhaustion in supplemented Difco Sporulation medium (DSM; Difco) [165], or by the resuspension method [164]. For the latter, *B*. *subtilis* cells were grown in a defined growth medium CH up to OD_600_ 0.6 prior to resuspension (T0) in the Sterlini-Mandelstam sporulation medium [163]. Optical density of the bacterial cultures was measured with NovaspecII Visible Spectrophotometer, Pharmacia Biotech. When required for selection, antibiotics were added at following concentrations: 100 *μ*g per ml of ampicillin, 100 *μ*g per ml of spectinomycin, 0.5 *μ*g per ml of erythromycin, 3 μg per ml of phleomycin, 5 μg per ml of kanamycin, and 5 *μ*g per ml of chloramphenicol. IPTG (isopropyl- β-D-1-thiogalactopyranoside) inducer was added to cells at concentrations indicated in the main text.

### Strains and plasmid construction

*E*. *coli* TG1 strain was used for plasmids construction. All *B*. *subtilis* strains were constructed at the basis of BSB1 strain (S6 Table). The used oligonucleotides are listed in S7 Table.

To construct the system for stable Rho expression, *rho* open reading frame was fused by PCR to the ribosome-binding site and spacer sequence of *B*. *subtilis tagD* gene using BSB1 chromosome as a template and oligonucleotides eb424 and eb458. The amplified fragment was cloned downstream P_*veg*_ promoter at pDG1730 plasmid using the blunted NheI and EagI sites. The resulting plasmid was transformed into *B*. *subtilis* BSB1 cells with selection for spectinomycin-resistance leading to the integration of the P*veg-rho* expression unit at chromosomal *amyE* locus by double crossover.

To construct a similar system expressing the tagged Rho, *rho-SPA* DNA fragment was amplified from the chromosome of BRL415 strain (S6 Table) using oligonucleotides eb458 and op148-R, digested by EagI endonuclease and cloned at pDG1730 between EagI and the filled-in NheI sites. The P*veg-rho*-SPA expression unit was inserted into the BSB1 chromosome as above.

To overexpress Rho in the strain BRL1250 containing P_*hyspank*_*-kinC* fusion (Spec^R^), P_*veg*_*-rho* expression unit was amplified from the chromosome of BRL802 strain using the oligonucleotides opv1730-B and veb596, digested by BamHI endonuclease and cloned between BamHI and blunted EcoRI sites of pSWEET vector. The resulting plasmid was used to reinsert the P_*veg*_-*rho* expression unit at the *amyE* locus of BSB1 strain as above with selection for chloramphenicol-resistance. The resulting strain BRL1248 was controlled for sporulation- and competence-negative phenotypes associated with Rho overexpression. Finally, P_*veg*_-*rho* fusion was transformed into BRL1250 cells with selection for chloramphenicol-resistance.

The *B*. *subtilis* partial loss-of-function mutants *guaB S121F* and *guaB T139I* were constructed as follows. The corresponding single-nucleotide mutations *c362t* and *c416t* (coordinates starting from the *guaB* +1 nucleotide) were introduced into the *guaB* gene by the two-step site-directed mutagenesis. First, the DNA fragments were PCR-amplified using the complementary mutagenic oligonucleotides veb852, veb853 (for *c362t*), and veb855, veb856 (for *c416t*) in pairs with correspondent primers veb857, veb858 (for *c362t*), and veb857, veb859 (for *c416t*). Next, the respective fragments were joined by PCR using the primers veb857 and veb858 (for *c362t*) and veb857 and veb859 (for *c416t*) and cloned at the thermo-sensitive shuttle plasmid pMAD between SalI and EcoRI sites. The resulting plasmids were transformed in BSB1 cells with the selection for erythromycin-resistance at non-permissive 37°C. In this way, the plasmids integrated at the chromosomal *guaB* locus by single crossover leading to *guaB* duplication. The selected clones were propagated without selection at permissive 30°C to induce plasmid replication and its segregation from the chromosome due to the recombination between the flanking *guaB* copies. The erythromycin-sensitive clones which lost the plasmid were tested for the presence of *guaB* mutations by PCR using common primer veb857 and the oligonuclotides veb851 and veb854, specific for *c362t* and *c416t* mutations, respectively. The selected *guaB* mutants were controlled by sequencing.

The strain containing a translational *comGA-gfp* fusion was constructed as follows: the *gfp* gene was PCR-amplified from the plasmid pCV0119 [165] using primers veb740 and veb741. A 695 bp chromosomal fragment containing the promoter and 5’UTR of the *comGA* gene was amplified by PCR using oligonucleotides veb917 and veb916, which 5’-end is complementary to veb740. Two fragments were joined by PCR using primers veb741 and veb917. The joined fragment was digested by EcoRI and SalI endonucleases and ligated with a similarly cut large fragment of the plasmid pUC18Cm-luc [71]. The resulting plasmid was transformed to *B*. *subtilis* BSB1 with selection for chloramphenicol resistance.

### Luciferase assay

Analysis of promoters’ activity using luciferase fusions was performed as described previously with minor modifications [71]. Cells were grown in LB medium to mid-exponential phase (optical density OD_600_ 0.4–0.5), after which cultures were centrifuged and resuspended to OD 1.0 in fresh DS media, to follow expression of *spo0A-luc* and s*poIIA-luc* fusions during sporulation, or in competence medium, to analyze *comK-luc* activity during competence development. Upon OD verification, these pre-cultures were next diluted in respective media to an OD_600_ 0.025. The starter cultures were distributed by 200μl in a 96-well black plate (Corning, USA) and Xenolight D-luciferin K-salt (Perkin, USA) was added to each well to final concentration 1.5 mg/mL. The cultures were incubated at 37°C with agitation and analyzed in Synergy 2 Multi-mode microplate reader (BioTek Instruments). Relative Luminescence Units (RLU) and OD_600_ were measured at 5 min intervals. Each fusion-containing strain was analyzed at least three times. Each experiment included four independent cultures of each strain.

### Epifluorescence microscopy, image processing and cell measurements

For all microscopic observations, cells were mounted on a 2% agarose pad and topped with a coverslip. Bacteria were imaged with an inverted microscope (Nikon Ti-E) equipped with an iLas2 laser coupling system from Roper Scientific (150 mW, 488nm and 50 mW, 561 nm), a 100× oil immersion phase objective, an ORCA-R2 camera (Hamamatsu) and controlled by the MetaMorph software package (v 7.8; Molecular Devices, LLC). The post-acquisition treatment of the images was done with the Fiji software [166–168]. Cultures of *B*. *subtilis* were performed as described above. To quantify morphological changes, cells were sampled during exponential growth (OD_600_ 0.2) and early stationary phase (OD_600_ 1.8), and mixed with the lipophilic fluorescent dye Nile Red (10 μg/ml final concentration), prior to observation by epifluorescence microscopy. The mean cell lengths were determined with the ChainTracer plugin of the Fiji software [169], on two independent experiments with N >140 (N_avg_ = 290).

To determine the frequency of cells entering into the sporulation pathway, cultures were sampled three hours after the induction of sporulation by the resuspension method [164]. Strains carrying an inducible *kinA* gene were supplemented with 0.1 mM IPTG at the time of resuspension. Sampled cells were mixed with the lipophilic fluorescent dye Mitotracker Red (10 μg/ml final concentration) prior to microscopic observation. Asymmetric septa were manually counted in two independent replicas (N > 450 per strain and per replica).

To assess the frequency of cells entering the competence state, cells bearing P_*comGA*_-*gfp* transcriptional fusion were grown in competence medium and sampled two hours after entering stationary phase (OD_600_ 1.5). The proportion of cells expressing the reporter GFP was determined by manual counting, in at least three fields of view and for a minimum of 600 cells per strain and per replica.

### Sporulation assay

For sporulation assay, cells were diluted in LB in a way to obtain the exponentially growing cultures after over-night incubation at 28°C. The pre-cultures were diluted in pre-warmed liquid DS medium at OD_600_ 0.025 and incubated at 37°C for 20 or 24 hours. To determine quantity of the spores, half of a culture was heated at 75°C for 15 min and cells from heated and non-heated samples were plated in sequential ten-fold dilutions at LB-agar plates. Colonies were counted after 36 h of incubation at 37°C, and the percentage of spores was calculated as the ratio of colonies forming units in heated and unheated samples. In the sporulation experiments employing the IPTG-inducible systems for *kinA* or *kinC* expression, cells were let to sporulate in the presence of IPTG at concentrations indicated in the text. Each experiment included three independent isogenic cultures. Four independent experiments were performed to establish sporulation efficiency of each strain.

### Genetic competence assay

To establish kinetics of competence development we used a two-step transformation procedure [164] with minor modifications. *B*. *subtilis* cells were grown in SpC medium to stationary phase (OD_600_ 1.5) and induced for competence by dilution 7-fold in SpII competence medium; at 30-min intervals, culture samples (0.25 ml) were mixed with *B*. *subtilis* BSF4217 genomic DNA (100 ng) or pIL253 plasmid DNA (500 ng), incubated for 30 min at 37°C and plated at LB plates containing erythromycin. Plates were incubated at 37°C for 18 h before colonies were counted. SpC is composed of a minimal salts medium MSM (14 g l^−1^ of K_2_HPO_4_, 6 g l^−1^ of KH_2_PO_4_, 1 g l^−1^ of C_6_H_5_Na_3_O_7_.2H_2_O and 2 g l^−1^ of (NH_4_)_2_SO_4_) supplemented with 0.5% (wt/vol) glucose, 6 mM MgSO_4_.7H_2_O, 0.2% (wt/vol) Yeast extract (Difco), 0.005% (wt/vol) L-tryptophan, and 0.01% (wt/vol) Casein hydrolisate (Oxoid). SpII medium is composed of MSM supplemented with 0.5% (wt/vol) glucose, 6 mM MgSO_4_.7H_2_O, 0.1% (wt/vol) Yeast extract (Difco), 0.0005% (wt/vol) L-tryptophan, and 0.005% (wt/vol) Casein hydrolisate (Oxoid).

### Western blotting

The crude cell extracts were prepared using Vibracell 72408 sonicator (Bioblock scientific). Bradford assay was used to determine total protein concentration in each extract. Equal amounts of total proteins were separated by SDS-PAGE (10% polyacrylamide). After the run, proteins were transferred to Hybond PVDF membrane (GE Healthcare Amersham, Germany), and the transfer quality was evaluated by staining the membrane with Ponceau S (Sigma-Aldrich). The SPA-tagged Rho protein was visualized by hybridization with the primary mouse ANTI-FLAG M2 monoclonal antibodies (Sigma-Aldrich; dilution 1:5,000) and the secondary goat peroxidise-coupled anti-mouse IgG antibodies A2304 (Sigma-Aldrich; dilution 1:20,000). The control Mbl protein was visualized using primary rabbit anti-Mbl antibodies (dilution 1:10,000) and the secondary goat peroxidase-coupled anti-rabbit IgG antibodies A0545 (Sigma-Aldrich; dilution 1:10,000). Three independent experiments were performed, and a representative result is shown in Fig 1B.

### ppGpp determination

To determine intracellular ppGpp level, *B*. *subtilis* cells were grown as described above to optical densities OD_600_ 0.5 (for argenine hydroxamate treatment analysis) or OD_600_ 1.5 (for stationary phase analysis). Bacterial cultures in triplicates (20 ml each) were rapidly centrifuged at 4°C and cellular pellets were frozen in liquid nitrogen. All extraction steps were performed on ice. Cellular pellets were deproteinized by addition of an equal volume of 6% perchloric acid (PCA) and incubation on ice for 10 min with two rounds of vortex-mixing for 20 s. Acid cell extracts were centrifuged at 13,000 rpm for 10 min at 4°C. The resulting supernatants were supplemented with an equal volume of bi-distilled water, vortex-mixed for 60 s, and neutralized by addition of 2 M Na_2_CO_3_. After filtration (3kDa cut off), extracts were injected onto a C18 Supelco 5 μm (250 × 4.6 mm) column (Sigma) at 45°C. The mobile phase was delivered using the stepwise gradient of buffer A (10 mM tetrabutylammonium hydroxide, 10 mM KH_2_PO_4_ and 0.25% MeOH; adjusted with 1M HCl to pH 6.9) and buffer B (5.6 mM tetrabutylammonium hydroxide, 50 mM KH_2_PO_4_ and 30% MeOH; adjusted with 1 M NaOH to pH 7.0) at a flow-rate of 1 ml/min and elution program: from 60%A + 40%B at 0 min to 40%A+60%B at 30 min and 40%A+60%B at 60 min.

Detection was done with a diode array detector (PDA). The LC Solution workstation chromatography manager was used to pilot the HPLC instrument and to process the data. Products were monitored spectrophotometrically at 254 nm, and quantified by integration of the peak absorbance area, employing a calibration curve established with various nucleoside standards. The ppGpp standard was purchased from Jena Bioscience GmbH (Germany). Finally, a correction coefficient was applied to correct raw data for minor differences in the densities of bacterial cultures.

### Transcriptome profiling by RNA sequencing

RNA was extracted from independent cultures of *B*. *subtilis* BSB1 WT, Δ*rho* and the Rho^+^ strains grown in LB medium at 37°C under vigorous agitation up to mid exponential or early stationary phase of growth (OD_600_ ~0.5 and ~2.0, respectively). Experiments were performed in duplicates for WT and mid-exponential samples and triplicates for early stationary phase of Δ*rho* and Rho^+^.

RNA preparation and DNase treatment were done as described [26]. Quality and quantity of RNA samples were analyzed on Bioanalyzer (Agilent, CA). The Next Generation Sequencing (NGS) Core Facility (Institute of Integrative Biology of the Cell, Gif-sur-Yvette, France; https://www.i2bc.paris-saclay.fr/sequencing/ng-sequencing/addon-ng-sequencing) prepared the RNAseq libraries with ScriptSeq protocol using RiboZero for rRNA-depletion (Illumina, San Diego, California) and generated strand-specific paired-end reads of 40 bp on an Illumina NextSeq platform (NextSeq 500/550 High Output Kit v2).

Reads were trimmed to remove adapters and low-quality ends using Cutadapt (v1.15, DOI:10.14806/ej.17.1.200) and Sickle (v1.33, options: -t sanger -x -n -q 20 -l 20) and mapped onto AL009126.3 reference genome assembly using Bowtie2 (v2.3.5.1; options "-N 1 -L 16 R 4", [170]. Counts of the number of read pairs (fragments) overlapping the sense and antisense strand of each transcribed region (AL009126.3-annotated genes and S-segments from [6] were obtained with Htseq-count (v0.11.0; options “-m union–nonunique = all”; [171].

Since Δ*rho*, WT and Rho^+^ exhibited different levels of pervasive transcription leading to global changes in low expression values and antisense signal; we selected a subset of well-expressed genes whose sense signal is in principle less impacted and thus most relevant for sequencing depth normalization. To this end, we selected the 728 AL009126.3-annotated genes satisfying, for all 4 WT samples, log2(fpkm_raw+5)>7, where fpkm_raw refers to the fpkm (fragments per kilobase of transcript per million mapped fragments) value obtained when library size is simply estimated of as the sum of counts. Differential gene expression analysis between conditions and strains, including sequencing depth normalization, was then conducted with R library "DESeq2" (v1.32.0; [172]). DESeq2 p-values for each pairwise comparison and each strand were converted into q-values using R library "fdrtool" (v1.2.17; [173]. Genes were called differentially expressed between strains or conditions when the estimated q-value ≤ 0.05 and the log2 fold change (|log2FC|) exceeded the cut-off specified in the text (0.5 or 1) for the considered strand (sense or antisense). Data was deposited in GEO (accession number GSE195579).

Graphical representations of the expression level of a gene in a given strain and condition used the geometrical mean of log2(fpkm+5) values, where FPKM was computed with the DESeq2-estimated library size factors multiplied by the median of sample count sums. To allow interactive exploration of the sense and antisense signal along the genome with bp-resolution, we also implemented in Genoscapist [95] the representation of a new data type corresponding to RNAseq coverage. For this purpose, count values are extracted with “bedtools genomecov” (version 2.27.0, 156; [174]) and represented as a step function with breakpoints corresponding to extremities of mapped read pairs along the genome sequence. To make these counts comparable between different samples and with gene-level expression values, coverage counts are converted to fpkm, using the formula fpkm_cov(t) = cov(t)*(10^3^/F)*(10^6^/L), where cov(t) is the coverage count for genome position t, L is the library size used to compute gene-level FPKM, and F is the average fragment length (from 178 bp to 200 bp across samples) obtained from the distance between extremities of inward oriented read pairs returned by "samtools stats" (version 1.10, 157.; [175]). The bp-level signal, displayed as log2(fpkm_cov(t)+5), can be accessed via the website http://genoscapist.migale.inrae.fr/seb_rho/.

### Preparation of protein samples and Mass spectrometry analysis

*B*. *subtilis* WT and Rho^+^ strains were grown in LB medium at 37°C agitation up to mid exponential or early stationary phase of growth (OD_600_ 0.5 and 2.0, respectively). All experiments were carried out in triplicate. Protein samples were prepared as described [26], 10 mg of total protein were loaded on the denaturing SDS-PAGE gel (10% AA) after heating for 10 min at 70°C with Laemmli loading buffer. After the gel separation proteins were visualized with SimpleBlue Safe Stain (Invitrogen, Coomassie G-250). In-gel digestion of the proteins was performed on bands excised from the SDS-PAGE. Each lane of migration was cut and washed for 15 min with an acetonitrile/100 mM ammonium bicarbonate mixture (1:1). Digestion was performed in 50 mM ammonium bicarbonate pH 8.0 and the quantity of modified trypsin (Promega, sequencing grade) was 0.1 μg per sample. Digestion was achieved for 6 h at 37°C. Peptides were extracted by 5% formic acid in water/acetonitrile (v/v). Supernatant and extracted tryptic peptides were dried and resuspended in 50 μL of of nano HPLC buffer containing 0.1% (v/v) formic acid and 2% (v/v) acetonitrile.

Mass spectrometry was performed on the PAPPSO platform (MICALIS, INRA, Jouy en Josas, France; http://pappso.inrae.fr/). An Orbitrap Fusion Lumos Tribrid (Thermo Fisher Scientific) coupled to an UltiMate 3000 RSLCnano System (Thermo Fisher Scientific) was used. A 4 μl sample was loaded at 20 μl/min on a precolumn (μ-Precolumn, 300 μm i.d x 5 mm, C18 PepMap100, 5 μm, 100 Å, Thermo Fisher) and washed with loading buffer. After 3 min, the precolumn cartridge was connected to the separating column (Acclaim PepMap, 75 μm x 500 mm, C18, 3 μm, 100 Å, Thermo Fisher). Buffer A consisted of 0.1% formic acid in 2% acetonitrile and buffer B of 0.1% formic acid in 80% acetonitrile. The peptide separation analysis was achieved at 300 nl/min with a linear gradient from 1 to 28% buffer B for 100 min and 28% to 40% for10 min. One run took 147 min, including the regeneration step at 98% buffer B. Ionization (1.6 kV ionization potential) and capillary transfer (275°C) were performed with a liquid junction and a capillary probe (SilicaTip Emitter, 10 μm, New Objective). Peptide ions were analyzed using Xcalibur 3.1.66.10. In HCD mode the machine settings were as follows: 1) full MS scan in Orbitrap (scan range [m/z] = 400–1500), resolution = 120 000 and 2) MS/MS using HCD (30% collision energy) in Orbitrap, resolution = 120 000, AGC target = 5.0 x 105, max. injection time = 50 ms, data type = profile. Analyzed charge states were set to 2–5, the dynamic exclusion to 80 s and the intensity threshold was fixed at 5.103. Protein identification was performed with X!Tandem software X!TandemPipeline (open source software developed by PAPPSO, version 3.4.3, http://pappso.inra.fr/bioinfo/xtandempipeline/) against a protein database of *B*. *subtilis* (version December 2019, 4188 entries). The proteins identification was run with a precursor mass tolerance of 10 ppm and a fragment mass tolerance of 20 ppm. Enzymatic cleavage rules were set to trypsin digestion (“after Arg and Lys, unless Pro follows directly after”) and no semi-enzymatic cleavage rules were allowed. The fix modification was set to cysteine carboxyamidomethylation and methionine oxidation was considered as a potential modification. In a second pass, N-terminal acetylation was added as another potential modification, whereas all other previous settings were retained. The identified proteins were filtered as follows: 1) peptide E value < 0.01 with a minimum of 2 peptides per protein and 2) a protein E-value of < 10–4. Peptide quantities of the proteome were analyzed by spectral counting (SC), peak counting and eXtracted Ion Current (XIC). Spectral counting takes into account the number of assigned spectra for each protein and is correlated to relative protein abundance. Peak counting analyses semi-quantitative variations between peaks (distinct peptide occurences), which are representative of protein abundance. For the quantification of peptides by XIC, MassChroQ version 0,4,4 [176] was used. The range for peak detection was set to 10 ppm with a detection threshold ranging from 3000 to 5000. All peptide intensities were log10 transformed for the following data treatments. The analysis using XIC requires the alignment of retention times, intensity normalization and log-transformation of values. Protein abundances are calculated as the sum of peptide intensities, which allows for analysis of quantitative variations [96,97]. Peptides with a variation ratio < 1.5 were eliminated, as well as peptides with a standard deviation from the retention time of 20 s and higher.

Proteins showing low numbers of spectra (< 5) in all the injections were eliminated. During the XIC analysis, the following data was eliminated additionally: peptides with a peak width higher than 200 s, peptides absent in more than 10% of samples and proteins quantified by a small number of peptides-modification-charge combination and peptides-mz showing too much variations of their retention time (40s). The data set was normalized based on the median RT and missing peptide intensities and protein abundances were imputed. The significance of variation was determined by an ANOVA (analysis of variance). The *p*-values obtained from ANOVA were considered significant below a value, respectively for the SC and the XIC: 0.05 and of 0.01.

## Supporting information

S1 Fig Graphical representation of the sporulation-proficient suppressor mutations in Rho from *B*. *subtilis* Rho^+^ strain. The primary sequence of *B*. *subtilis* Rho subunit is shown (NP_391589.2). The major characteristic motifs identified previously by studies of different Rho proteins [177, 178] are boxed and highlighted in grey. The amino acid substitutions identified in Rho^+^ suppressors are marked in red. Three point mutations might have drastic effect on Rho activity. Replacement of glycine by arginine at the positions 286 and 287 (G286R and G287R, respectively) could destroy the highly conserved Q-loop forming a secondary RNA binding site, while the substitution of alanine177 localized within one of the Walker motifs by threonine (A177T; isolated twice) could affect ATP binding. Indeed, as shown in a complementation assay (S2 Table), suppressor mutations G287R, G286R and A177T completely inactivate Rho protein, while N274H (isolated twice) and P335R mutant proteins remain partially active. Two mutant Rho proteins were truncated by a stop codon at the position 146 (Q146Stop).
(TIF)

S2 Fig Synthetic over-production of sensor histidine kinase KinA partially rescues an altered commitment to sporulation of Rho^+^ cells. *B*. *subtilis* WT and Rho^+^ strains and their respective derivatives expressing *kinA* gene under the control of the IPTG-inducible promoter P_*hyspank*_ were induced to sporulate by resuspension of the actively growing cultures in a minimal Sterlini-Mandelstam medium (Materials and methods). To induce *kinA* expression, the strains carrying P_*hyspank*_-*kinA* fusion were supplemented with 0.1 mM IPTG at the time of resuspension. Three hours after resuspension the cultures were analyzed by microscopy (**A**) and cells containing asymmetric septum or forespore were manually counted in two independent replicas (N > 450 per strain and per replica) (**B**). The experiment was reproduced twice and the results of a representative experiment are shown.
(TIF)

S3 Fig *B*. *subtilis* Rho^+^ exhibits competence–negative phenotype. Transformability of *B*. *subtilis* WT (blue lines) and Rho^+^ (red lines) strains by the plasmid pIL253 (filled-in circles) and homologous genomic DNA (opened circles). Competence induction and transformation were performed as described in Materials and Methods and Fig 3B. The experiment included three biological replicas of each strain and was reproduced twice. The results of a representative experiment are presented. The data are independent from Fig 3B.
(TIF)

S4 Fig Activity of the P_*comK*_ promoter in Rho^+^ cells. (**A**) The Rho^+^ strain differs from *spo0A* mutant in the activation of *comK*. Kinetics of luciferase expression in *B*. *subtilis* WT (blue line), Rho^+^ (red line) and *spo0A* (gray line with squares) mutant cells bearing the P_*comK*_*-luc* transcription fusion and grown in competence-inducing medium as described in Materials and Methods. (**B, C, D**) Inactivation of CodY, AbrB and Rok suppressors differently affects *comK* expression in *B*. *subtilis* WT and Rho^+^ cells. Kinetics of luciferase expression in WT P_*comK*_*-luc* and Rho^+^ P_*comK*_*-luc* cells mutated for: (**B**) *codY* (brown lines with filled-in and opened circles for WT and Rho^+^, respectively), (**C**) *abrB* (green lines with filled-in and opened triangles for WT and Rho^+^, respectively), and (**D**) *rok* (light-blue lines with filled-in and opened squares for WT and Rho^+^, respectively), and grown as in (A). The indicated mutant pairs were analyzed in parallel with the control parental strains WT P_*comK-luc*_ (blue line) and Rho^+^ P_*comK-luc*_ (red line). In (**A** and **B**), characteristic growth kinetics of WT and Rho^+^ cells (A) and their *codY* derivatives (B) are depicted by the respectively colored double-lined curves. In (**A-D**), data acquisition and processing were performed as in Fig 3C. For each strain, plotted are the mean values of luminescence readings corrected for OD from four independent cultures analyzed simultaneously. Each strain was analyzed at least three times. The results of representative experiments are shown.
(TIF)

S5 Fig Genome wide effects of Rho over-production on the *B*. *subtilis* transcriptome during exponential growth and stationary phase in rich medium. Scatter plots display the transcriptome changes in the antisense (**A** and **B**) and sense (**C** and **D**) strands by comparing *B*. *subtilis* Rho^+^ and WT strains during exponential growth (**A** and **C**) and stationary phase (**B** and **D**), respectively. Horizontal and vertical dashed lines correspond to the cut-off for minimal expression at log2(fpkm+5)≥5, as in Fig 6. The diagonal median solid line indicates unchanged expression levels, while dashed and dotted lines delineate DE changes of the |log2FC|≥1 and |log2FC|≥2, respectively. Each point represents one of the 4,292 AL009126.3-annotated genes. Point coordinates on x- and y-axes correspond to the normalized expression level (average of log2(fpkm+5) over biological replicates) measured with RNAseq in *B*. *subtilis* WT and Rho^+^, respectively. Background colors of the points indicate TRs whose transcription level is strongly up-regulated (yellow) or down-regulated (blue) in the Rho^+^ vs. WT comparison.
(TIF)

S6 Fig Differential expression of the ComK, CodY and stringent response regulons in *B*. *subtilis* Δ*rho* strain. Scatter plots display the expression values for the each gene from the ComK regulon (**A** and **B**), CodY and the stringent response regulon (**C** and **D**) by comparing *B*. *subtilis* Δ*rho* and WT strains under conditions of exponential growth (**A, C**) and stationary phase (**B, D**). Each symbol represents one of the 4,292 AL009126.3-annotated genes. Colored triangles indicate the genes from ComK (red), CodY (blue) and the stringent response (orange) regulons, respectively; gray circles represent genes outside of the analyzed regulons. Expression levels are represented on x- and y-axes by the mean log2(fpkm+5) between biological replicates as in Fig 6. Horizontal and vertical dashed lines correspond to the cut-off for minimal expression at log2(fpkm+5)≥5. Genes near the central diagonal (solid line) have unchanged expression between strains whereas dashed and dotted diagonal lines indicate approximately |log2FC|≥1 and |log2FC|≥2. The genes activated or repressed by cognate regulator are denoted as in Fig 6. Gene names mentioned in the text are indicated.
(TIF)

S7 Fig Thermo-sensitivity of the Rho^+^ strain. (**A**) Lowering GTP levels does not rescue thermo-sensitive phenotype of the Rho^+^ strain. *B*. *subtilis* WT, Rho^+^ cells and their respective *guaB* S121F and *guaB* T139I mutants were grown in LB medium at 37°C to mid exponential phase (OD_600_ 0.5), spotted in serial dilutions on LB agar plates and incubated at 37°C and 55°C for 18 hours. (**B**) Thermo-sensitivity of the Rho^+^ strain is not due to a low level of *comGA* expression, as *comGA* mutant resists high temperature. *B*. *subtilis* WT, its isogenic *comK* and *comGA* mutants and Rho^+^ cells growing exponentially (OD_600_ 0.5) in LB medium were streaked on LB agar plates and incubated at 37°C and 55°C for 18 hours before imagining. The experiments were reproduced at least three times and the representative results are shown.
(TIF)

S1 Table Proteomic analysis of *B*. *subtilis* WT and Rho^+^ strains grown in LB medium. (XLSX)

S2 Table Sporulation proficient and thermo-resistant suppressors of the Rho^+^ strain. (PDF)

S3 Table Differential expression analysis of Rho^+^ and Δ*rho* vs. WT *B*. *subtilis* BSB1 cells. (XLSX)

S4 Table S-segments directly regulated by Rho-dependent transcription terminators. (XLSX)

S5 Table Comparison between sets of DE genes and *Subti*Wiki regulons. (XLSX)

S6 Table Strains and plasmids used in this study. (DOCX)

S7 Table Oligonucleotides used for strains construction. (DOCX)

S8 Table Source data for figures. (XLSX)
