# Supplementary figures and images for: Termination factor Rho mediates transcriptional reprogramming of Bacillus subtilis stationary phase
Source: PLoS Genet. 2023 Feb 3;19(2):e1010618. doi: 10.1371/journal.pgen.1010618 (PMC9931155; doi:10.1371/journal.pgen.1010618)

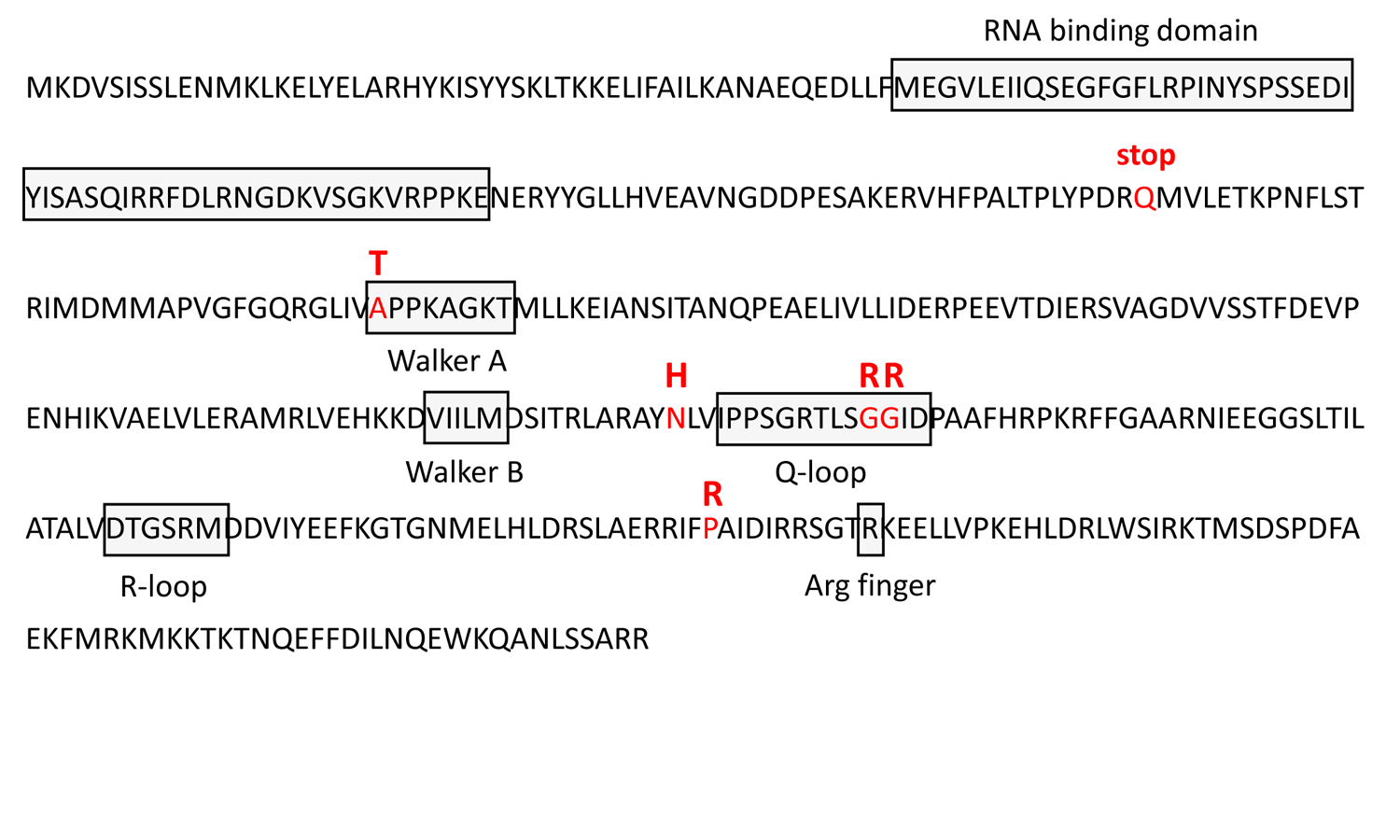

Supplement: S1 Fig — The primary sequence of B. subtilis Rho subunit is shown (NP_391589.2). The major characteristic motifs identified previously by studies of different Rho proteins [177, 178] are boxed and highlighted in grey. The amino acid substitutions identified in Rho+ suppressors are marked in red. Three point mutations might have drastic effect on Rho activity. Replacement of glycine by arginine at the positions 286 and 287 (G286R and G287R, respectively) could destroy the highly conserved Q-loop forming a secondary RNA binding site, while the substitution of alanine177 localized within one of the Walker motifs by threonine (A177T; isolated twice) could affect ATP binding. Indeed, as shown in a complementation assay (S2 Table), suppressor mutations G287R, G286R and A177T completely inactivate Rho protein, while N274H (isolated twice) and P335R mutant proteins remain partially active. Two mutant Rho proteins were truncated by a stop codon at the position 146 (Q146Stop). (TIF) [file pgen.1010618.s001.tif]

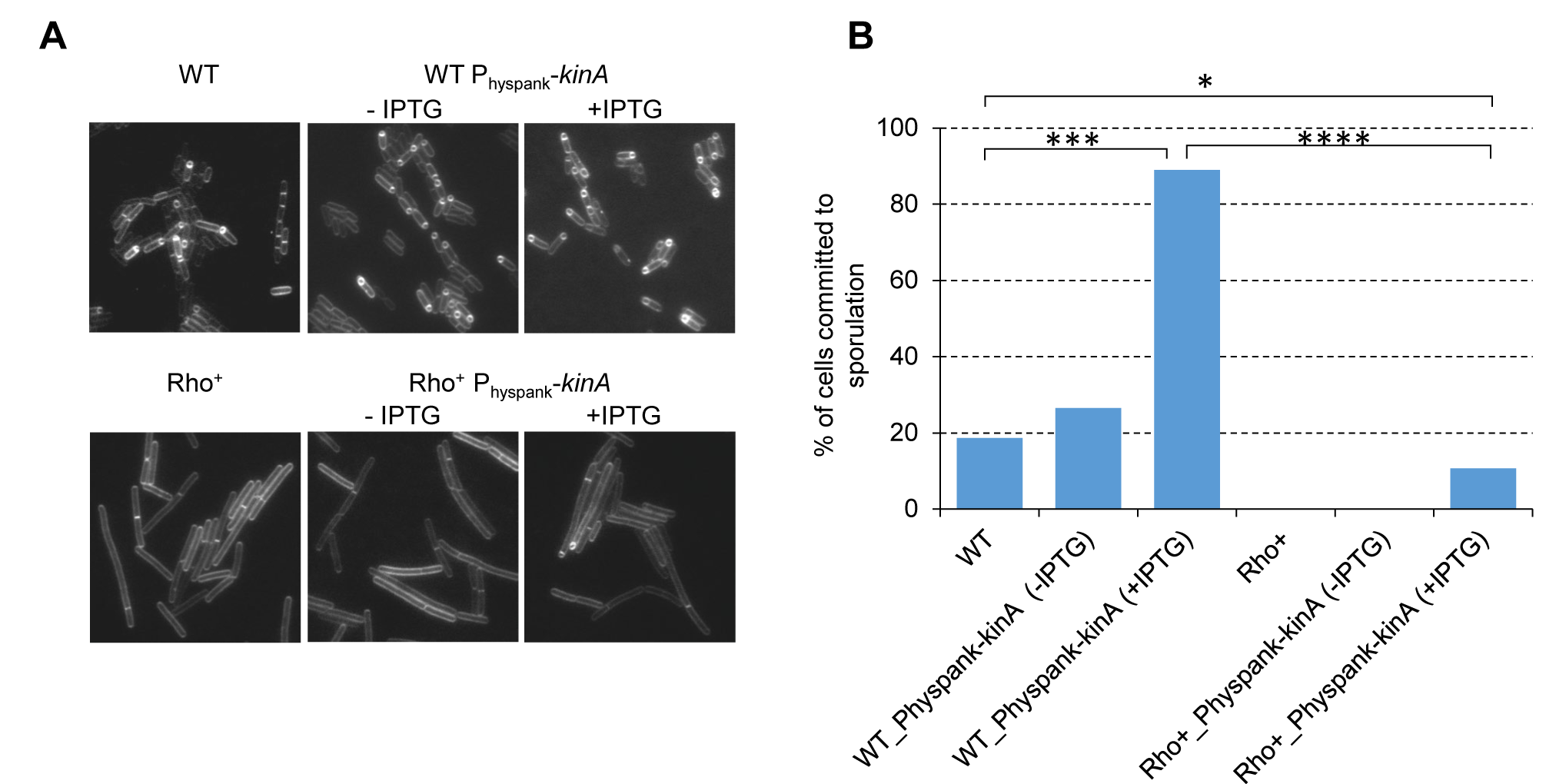

Supplement: S2 Fig — B. subtilis WT and Rho+ strains and their respective derivatives expressing kinA gene under the control of the IPTG-inducible promoter Physpank were induced to sporulate by resuspension of the actively growing cultures in a minimal Sterlini-Mandelstam medium (Materials and methods). To induce kinA expression, the strains carrying Physpank-kinA fusion were supplemented with 0.1 mM IPTG at the time of resuspension. Three hours after resuspension the cultures were analyzed by microscopy (A) and cells containing asymmetric septum or forespore were manually counted in two independent replicas (N > 450 per strain and per replica) (B). The experiment was reproduced twice and the results of a representative experiment are shown. (TIF) [file pgen.1010618.s002.tif]

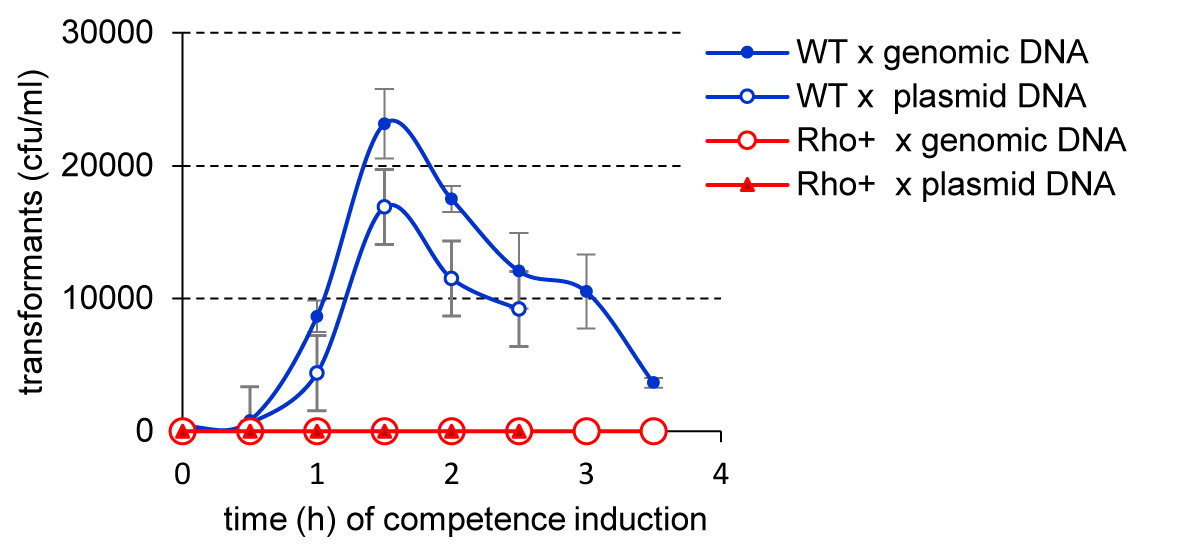

Supplement: S3 Fig — Transformability of B. subtilis WT (blue lines) and Rho+ (red lines) strains by the plasmid pIL253 (filled-in circles) and homologous genomic DNA (opened circles). Competence induction and transformation were performed as described in Materials and Methods and Fig 3B. The experiment included three biological replicas of each strain and was reproduced twice. The results of a representative experiment are presented. The data are independent from Fig 3B. (TIF) [file pgen.1010618.s003.tif]

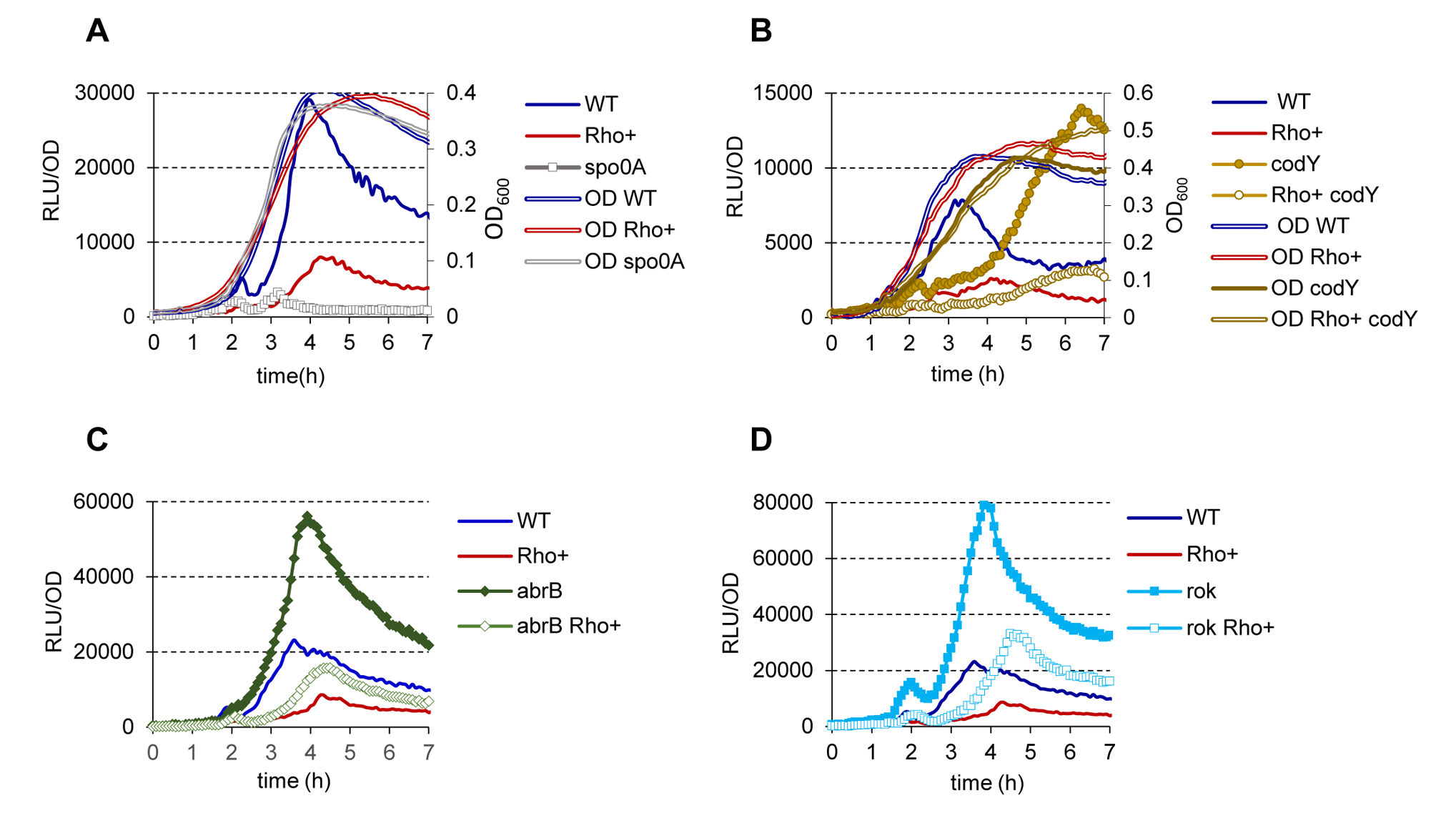

Supplement: S4 Fig — (A) The Rho+ strain differs from spo0A mutant in the activation of comK. Kinetics of luciferase expression in B. subtilis WT (blue line), Rho+ (red line) and spo0A (gray line with squares) mutant cells bearing the PcomK-luc transcription fusion and grown in competence-inducing medium as described in Materials and Methods. (B, C, D) Inactivation of CodY, AbrB and Rok suppressors differently affects comK expression in B. subtilis WT and Rho+ cells. Kinetics of luciferase expression in WT PcomK-luc and Rho+ PcomK-luc cells mutated for: (B) codY (brown lines with filled-in and opened circles for WT and Rho+, respectively), (C) abrB (green lines with filled-in and opened triangles for WT and Rho+, respectively), and (D) rok (light-blue lines with filled-in and opened squares for WT and Rho+, respectively), and grown as in (A). The indicated mutant pairs were analyzed in parallel with the control parental strains WT PcomK-luc (blue line) and Rho+ PcomK-luc (red line). In (A and B), characteristic growth kinetics of WT and Rho+ cells (A) and their codY derivatives (B) are depicted by the respectively colored double-lined curves. In (A-D), data acquisition and processing were performed as in Fig 3C. For each strain, plotted are the mean values of luminescence readings corrected for OD from four independent cultures analyzed simultaneously. Each strain was analyzed at least three times. The results of representative experiments are shown. (TIF) [file pgen.1010618.s004.tif]

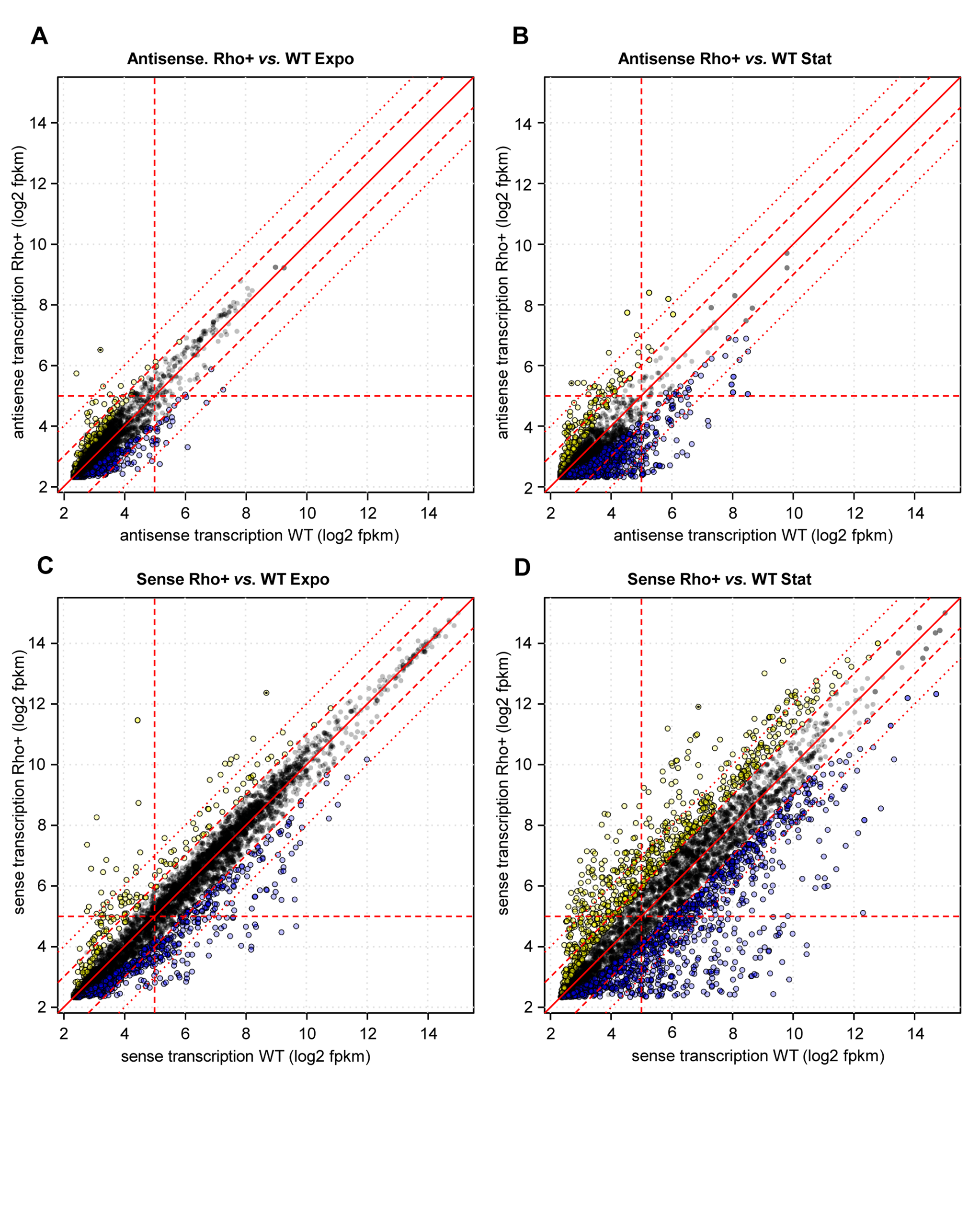

Supplement: S5 Fig — Scatter plots display the transcriptome changes in the antisense (A and B) and sense (C and D) strands by comparing B. subtilis Rho+ and WT strains during exponential growth (A and C) and stationary phase (B and D), respectively. Horizontal and vertical dashed lines correspond to the cut-off for minimal expression at log2(fpkm+5)≥5, as in Fig 6. The diagonal median solid line indicates unchanged expression levels, while dashed and dotted lines delineate DE changes of the |log2FC|≥1 and |log2FC|≥2, respectively. Each point represents one of the 4,292 AL009126.3-annotated genes. Point coordinates on x- and y-axes correspond to the normalized expression level (average of log2(fpkm+5) over biological replicates) measured with RNAseq in B. subtilis WT and Rho+, respectively. Background colors of the points indicate TRs whose transcription level is strongly up-regulated (yellow) or down-regulated (blue) in the Rho+ vs. WT comparison. (TIF) [file pgen.1010618.s005.tif]

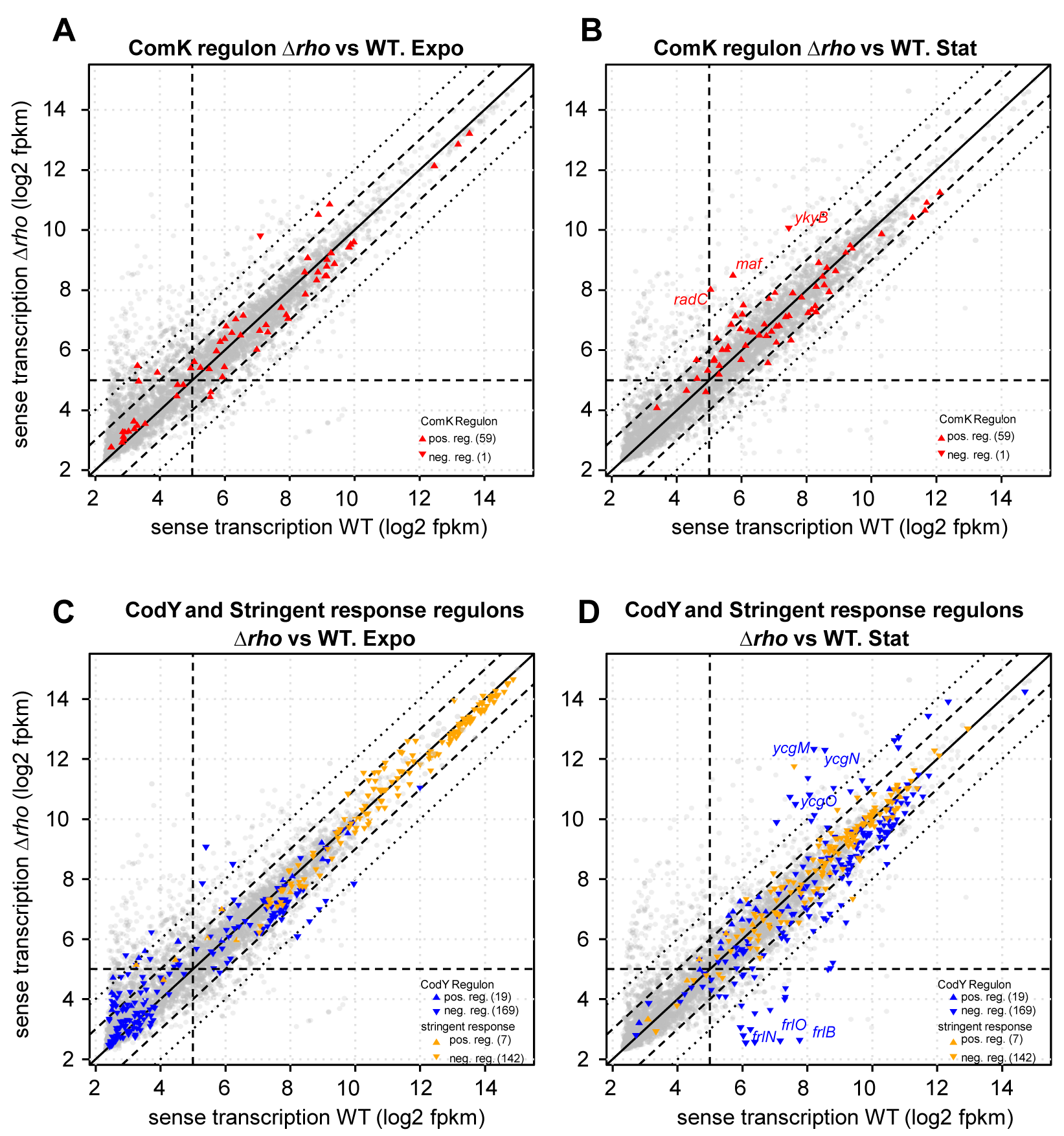

Supplement: S6 Fig — Scatter plots display the expression values for the each gene from the ComK regulon (A and B), CodY and the stringent response regulon (C and D) by comparing B. subtilis Δrho and WT strains under conditions of exponential growth (A, C) and stationary phase (B, D). Each symbol represents one of the 4,292 AL009126.3-annotated genes. Colored triangles indicate the genes from ComK (red), CodY (blue) and the stringent response (orange) regulons, respectively; gray circles represent genes outside of the analyzed regulons. Expression levels are represented on x- and y-axes by the mean log2(fpkm+5) between biological replicates as in Fig 6. Horizontal and vertical dashed lines correspond to the cut-off for minimal expression at log2(fpkm+5)≥5. Genes near the central diagonal (solid line) have unchanged expression between strains whereas dashed and dotted diagonal lines indicate approximately |log2FC|≥1 and |log2FC|≥2. The genes activated or repressed by cognate regulator are denoted as in Fig 6. Gene names mentioned in the text are indicated. (TIF) [file pgen.1010618.s006.tif]

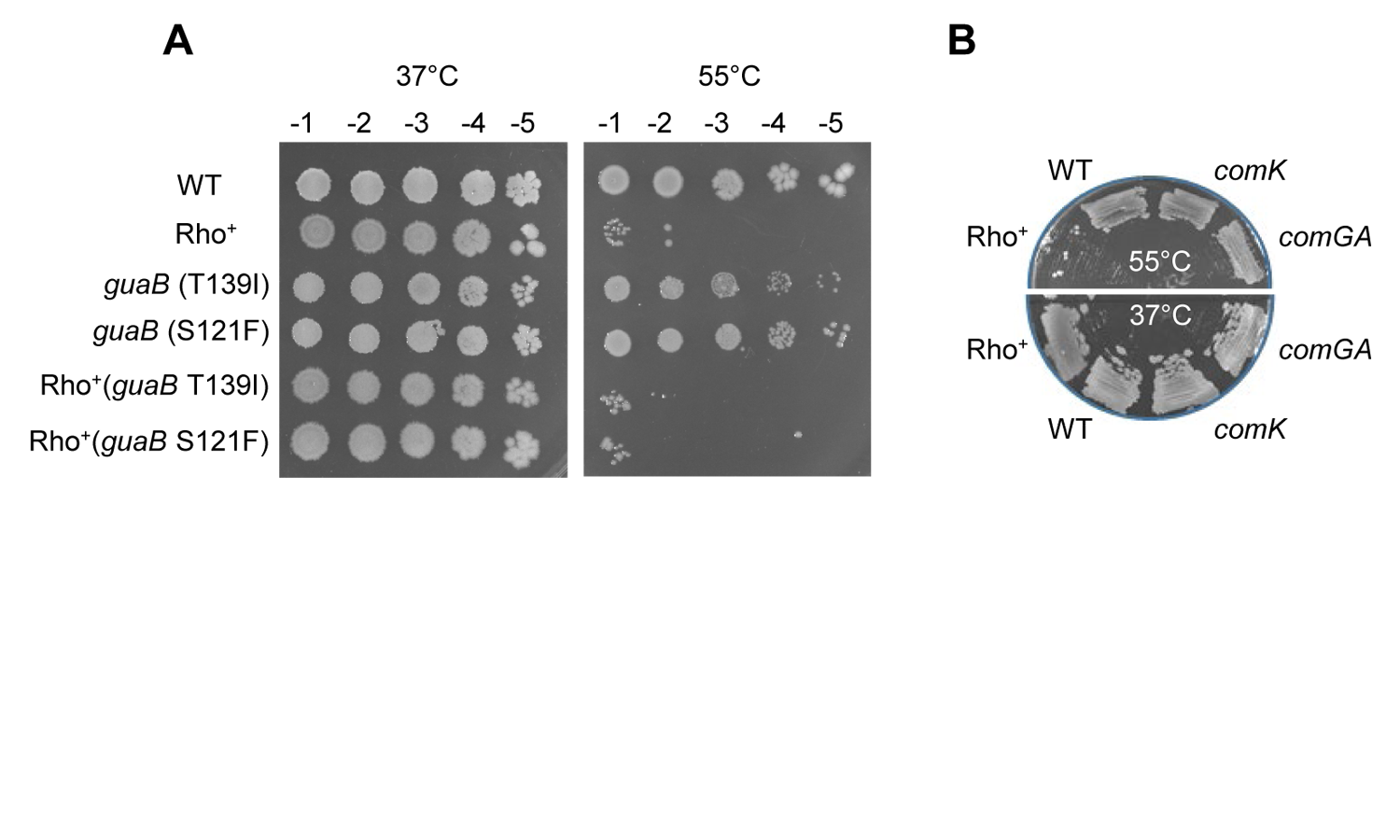

Supplement: S7 Fig — (A) Lowering GTP levels does not rescue thermo-sensitive phenotype of the Rho+ strain. B. subtilis WT, Rho+ cells and their respective guaB S121F and guaB T139I mutants were grown in LB medium at 37°C to mid exponential phase (OD600 0.5), spotted in serial dilutions on LB agar plates and incubated at 37°C and 55°C for 18 hours. (B) Thermo-sensitivity of the Rho+ strain is not due to a low level of comGA expression, as comGA mutant resists high temperature. B. subtilis WT, its isogenic comK and comGA mutants and Rho+ cells growing exponentially (OD600 0.5) in LB medium were streaked on LB agar plates and incubated at 37°C and 55°C for 18 hours before imagining. The experiments were reproduced at least three times and the representative results are shown. (TIF) [file pgen.1010618.s007.tif]
