# Supplementary material for: Termination factor Rho mediates transcriptional reprogramming of Bacillus subtilis stationary phase
Source: PLoS Genet. 2023 Feb 3;19(2):e1010618. doi: 10.1371/journal.pgen.1010618 (PMC9931155; doi:10.1371/journal.pgen.1010618)
Supplement: S2 Table — (PDF) [file pgen.1010618.s009.pdf]

S2 Table **Sporulation proficient and thermo-resistant suppressors of Rho^+ strain.**

1. Sporulation proficient suppressors

	sporulation efficiency(*)			structural analysis of the mutant RhoEU		propeties of the mutant Rho proteins(****)	
	A	B	C	D	E	F	G
	initial isolate	RhoEU(**) transfered to RM (*Δrho*)	RhoEU transfered to WT	nucleotide change (***)	amino acid change	*Δrho* complementation/ activity	relationship with the wild type Rho
Rho+ supp1	6,54E-01	8,57E+00	1,52E+00	c436t	Gln146Stop	no/inactive	recessive
Rho+ supp2	3,49E-01	8,53E-01	8,44E-01	c1064g	Pro335Arg	yes/partially active	recessive
Rho+ supp3	2,80E+00	5,26E+00	4,02E+00	g856a	Gly286Arg	no/inactive	negatively dominant
Rho+ supp4	4,42E-01	9,85E+00	6,93E-01	c436t	Gln146Stop	no/inactive	recessive
Rho+ supp5	6,81E-01	1,09E+00	1,19E+00	a820c	Asn274His	yes/partially active	recessive
Rho+ supp6	4,18E+00	3,86E+00	5,17E+00	g859a	Gly287Arg	no/inactive	negatively dominant
Rho+ supp7	4,97E+00	4,74E+00	5,34E+00	g529a	Ala177Thr	no/inactive	negatively dominant
Rho+ supp8	1,12E+00	1,26E+00	1,40E+00	a820c	Asn274His	yes/partially active	recessive
**controls**							
WT	1,00E+00						
RM (*Drho*)	1,02E+01						
Rho+	2,87E-04						

(*) all data were obtained in the same experiment
(**) Rho over-expression unit from the initial isolate
(***) Position of the mutation from the +1 nucleotide of *rho* gene
(****) see Table Legend for details

2. Termo-resistant suppressors

	DNA mutation (*): nucleotide deletion or insertion	amino acid change / Rho protein modification
Rho+ suppTR1	insertion of adenine at 1207	Thr403Asn / 39 novel C-terminal amino acids
Rho+ suppTR2	insertion of adenine at 100	Thr36Arg / Stop at aa64
Rho+ suppTR3	deletion of guanine at 932	Ser311Thr / Stop at aa312
Rho+ suppTR4	deletion of guanine at 1200	Met400Ile / 34 novel C-terminal amino acids

1. Sporulation proficient suppressors

All strains were tested for sporulation in two independent experiments, which gave very similar results. The data of the representative experiment are shown. For simplicity, the sporulation efficiencies in colons *A, B* and *C* are normalized to the WT control. (**A**) Eight independent clones, which had been isolated as heat-resistant spores from the Rho^+ cultures in DS medium, were compared for the efficiency of sporulation with the WT, RM (*Δrho*) and Rho^+ strains in order to confirm that they appeared due to a mutation. All candidate clones showed significantly higher levels of sporulation than the parental Rho^+ strain, indicating their mutant authenticity. (**B and C**) To understand whether or not a mutation was associated with the Rho over-expression unit (RhoEU), the RM (**B**) and WT (**C**) strains were transformed by the mutant chromosomal DNAs with selection for resistance to spectinomycin (the marker linked to RhoEU) and tested for sporulation efficiency. If the mutation was localized outside RhoEU, the transformants would have been inhibited for sporulation similarly to Rho^+ cells. However, all transformants sporulated significantly more efficiently than Rho^+ strain, indicating that the mutations disabled RhoEU. (**D and E**) Sequencing of the mutant RhoEUs identified the one-nucleotide substitutions within the *rho* open reading frame. The mutations modify the Rho proteins by single amino acid change. (**F and G**) The data presented in *B* and *C* provide some insights into the properties of the mutant Rho proteins. (**F**) In RM cells, inhibition of the high sporulation phenotype by the mutant RhoEU (supp2, and supp5, 8) indicates complementation of the *rho* deletion, which signifies that the mutant Rho remains partially active. (**G**) In WT cells, an increased sporulation in the presence of the mutant RhoEU (supp3, 6 and 7) indicates a decreased activity of the wild type Rho protein expressed from natural locus, which characterizes the mutations as dominant negative [1]. We infer that a mutant Rho remains able to oligomerize and, when overexpressed, forms inactive hexamers with the wild-type Rho monomers. In the opposite case, the mutation is recessive to the wild type *rho*.

2. Thermo-resistant suppressors

As described in the Text and shown at Fig 9, the cells over-expressing Rho are sensitive to 55°C, but produce few thermo-resistant (TR) clones when spotted at LB agar. Sequence analysis of RhoEUs from four independent TR clones revealed single nucleotide deletions or insertions within the rho open reading frame. This suggests different mechanisms of rho mutagenesis during sporulation and at high growth temperature.

Reference

1. Herskowitz I. Functional inactivation of genes by dominant negative mutations. Nature. 1987; 329: 219 -222. doi: 10.1038/329219a0.
