# Supplementary material for: Termination factor Rho mediates transcriptional reprogramming of Bacillus subtilis stationary phase
Source: PLoS Genet. 2023 Feb 3;19(2):e1010618. doi: 10.1371/journal.pgen.1010618 (PMC9931155; doi:10.1371/journal.pgen.1010618)
Supplement: S6 Table — (DOCX) [file pgen.1010618.s013.docx]

**S6 Table. Strains and plasmids used in this study.**

| **Strains** | **Genotype (resistance)** | **Source or reference** |
| --- | --- | --- |
| BSB1 | *B. subtilis* 168 *trp^+^* | [1] |
| BRL1 | BSB1 *Δrho::phleo* | [2] |
| BRL802  (Rho^+^ spec) | BSB1 *amyE::Pveg-rho* (Sp^R^) | This study |
| BRL415 | BSB1 *rho-SPA* (Em^R^) | [2] |
| BRL796 | BSB1 *amyE::Pveg-rho-SPA* (Sp^R^) | This study |
| BRL116 | BSB1 P*spo0A-luc* (Cm^R^) | [2] |
| BRL831 | Rho^+^ P*spo0A-luc* (Cm^R^ Sp^R^) | This study |
| BKK00980 | *B. subtilis* 168 *trpC2* *spo0H::kan* (Km^R^) | [3] |
| BRL896 | BSB1 *spo0H::kan* P*spo0A-luc* | This study |
| BRL111 | BSB1 P*spoIIA-luc* (Cm^R^) | [2] |
| BRL809 | Rho^+^  P*spoIIA-luc* (Cm^R^) | This study |
| MF1913 | *B. subtilis* PY79 P_hyspank_-kinA (CmR) | [4] |
| BRL1240 | BSB1 P_hyspank_-kinA (Cm^R^) | This study |
| BRL1241 | Rho+ P_hyspank_-kinA (CmR Sp^R^) | This study |
| RL3606 | *B. subtilis* PY79 kinCΩP_hyspank_-kinC (Sp^R^) | [5] |
| BRL1244 | BSB1 kinCΩP_hyspank_-kinC (Sp^R^) | This study |
| BRL1248  (Rho^+^ cat) | BSB1 *amyE::Pveg-rho* (Cm^R^) | This study |
| BRL1250 | Rho^+^ kinCΩP_hyspank_-kinC (Sp^R^Cm^R^) | This study |
| BD4773 | *B. subtilis* P*comK-luc* (Cm^R^) | [6] |
| BRL115 | BSB1 P*comK-luc* (Cm^R^) | This study |
| BRL829 | Rho^+^ P*comK-luc* (Sp^R^ Cm^R^) | This study |
| BKK16170 | *B. subtilis* 168 *trpC2 codY::kan* (Km^R^) | [3] |
| BRL1048 | BSB1 *codY::kan* (Km^R^) | This study |
| BRL1050 | Rho^+^ *codY::kan* (Km^R^ Sp^R^) | This study |
| BRL1126 | BSB1 P*comK-luc* *codY::kan* (Cm^R^ Km^R^) | This study |
| BRL1133 | Rho^+^ P*comK-luc* *codY::kan* (Cm^R^ Km^R^ Sp^R^) | This study |
| BKK14240 | *B. subtilis* 168 *trpC2 rok::kan* (Km^R^) | [3] |
| BRL1273 | BSB1 *rok::kan* (Km^R^) | This study |
| BRL1274 | Rho^+^ *rok::kan* (Km^R^ Sp^R^) | This study |
| BRL1303 | BSB1 P*comK-luc* *rok::kan* (Cm^R^ Km^R^) | This study |
| BRL1304 | Rho^+^ P*comK-luc* *rok::kan* (Cm^R^ Km^R^ Sp^R^) | This study |
| BMR50 | *B.subtilis* 168 *abrB::phleo* (Pm^R^) | [7] |
| BRL1338 | BSB1 *abrB::phleo* (Pm^R^) | This study |
| BRL1339 | Rho^+^ *abrB::phleo* (Pm^R^ Sp^R^) | This study |
| BRL1335 | BSB1 P*comK-luc* *abrB::phleo* (Cm^R^ Pm^R^) | This study |
| BRL1336 | Rho^+^ P*comK-luc* *abrB::phleo* (Cm^R^ Pm^R^ Sp^R^) | This study |
| BRL1340 | BSB1 *abrB::phleo* *rok::kan* (Km^R^ Pm^R^) | This study |
| BRL1341 | Rho^+^ *abrB::phleo* *rok::kan* (Km^R^ Pm^R^ Sp^R^) | This study |
| BRL1342 | BSB1 P*comK-luc* *abrB::phleo* *rok::kan* (Cm^R^ Km^R^ Pm^R^) | This study |
| BRL1343 | Rho^+^ P*comK-luc* *abrB::phleo* *rok::kan* (Cm^R^ Km^R^ Pm^R^ Sp^R^) | This study |
| BRL1370 | WT P_comGA_-gfp (Cm^R^) | This study |
| BRL1373 | Rho^+^ P_comGA_-gfp (Cm^R^ Sp^R^) | This study |
| BRL1376 | WT P_comGA_-gfp *abrB::phleo* *rok::kan* (Cm^R^ Pm^R^ Km^R^) | This study |
| BRL1378 | Rho^+^ P_comGA_-gfp *abrB::phleo* *rok::kan* (Cm^R^ Pm^R^ Km^R^ Sp^R^) | This study |
| BRL1267 | BSB1 *guaBS121F* | This study |
| BRL1268 | BSB1 *guaBT139I* | This study |
| BRL1271 | Rho^+^ *guaBS121F* (Sp^R^) | This study |
| BRL1272 | Rho^+^ *guaBT139I* (Sp^R^) | This study |
| CCB1050 *(p)ppGpp^0^* | *B. subtilis* W168 *yjbM::spc ywaC::kan relA::ery* (Em^R^ Km^R^ Sp^R^) | [8, 9] |
|  | | |
| **Plasmids** |  |  |
| pMAD | Erm^R^, Amp^R^ | [10] |
| pDG1730 | Sp^R^, Amp^R^ | [11] |
| pSWEET | Cm^R^, Amp^R^ | [12] |
| pUC18Cm-luc | Cm^R^, Amp^R^ | [13] |
| pDG1730-rho | Pveg-rho at pDG1730 ; Sp^R^, Amp^R^; used to construct BRL802 | This study |
| pDG1730-rho-SPA | Pveg-rho-SPA  at pDG1730 ; Sp^R^, Amp^R^; used to construct BRL796 | This study |
| pSWEET-Pveg-rho | Pveg-rho at pSWEET ; Cm^R^, Amp^R^; used to construct BRL1248 | This study |
| pMAD*guaBS121F* | Erm^R^, Amp^R^; used to construct BRL1267 | This study |
| pMAD*guaBS139I* | Erm^R^, Amp^R^; used to construct BRL1268 | This study |
| pUCm-comGA-gfp | PcomGA-gfp at pUC18Cm-luc; Cm^R^, Amp^R^; used to construct BRL1370 | This study |

**References**

1. Nicolas P, Mäder U, Dervyn E, Rochat T, Leduc A, Pigeonneau N, et al. Condition-dependent transcriptome reveals high-level regulatory architecture in Bacillus subtilis. Science. 2012; 335: 1103-1106.
2. Bidnenko V, Nicolas P, Grylak-Mielnicka A, Delumeau O, Auger S, Aucouturier A, et al. Termination factor Rho: from the control of pervasive transcription to cell fate determination in Bacillus subtilis. PLoS Genet. 2017; 13(7): e1006909.
3. Koo BM, Kritikos G, Farelli JD, Todor H, Tong K, Kimsey H, et al. Construction and analysis of two genome-scale deletion libraries for *Bacillus subtilis*. Cell Systems. 2017; 4: 291-305.
4. Fujita M, Losick R. Evidence that entry into sporulation in *Bacillus subtilis* is governed by a gradual increase in the level and activity of the master regulator Spo0A. Genes Dev. 2005; 19: 2236-2244.
5. Fujita M, González-Pastor JE, Losick R. High-and low-threshold genes in the Spo0A regulon of Bacillus subtilis. J Bacteriol. 2005; 187: 1357-1368.
6. Mirouze N, Desai Y, Raj A, Dubnau D. Spo0A~P imposes a temporal gate for the bimodal expression of competence in *Bacillus subtilis*. PLoS Genet. 2012; 8(3): e1002586.
7. KobirA, Poncet S, Bidnenko V, Delumeau O, Jers C, Zouhir S, et al. Phosphorylation of *Bacillus subtilis* AbrB. Mol Microbiol. 2014; 92: 1129-1141.
8. Trinquier A, Ulmer JE, Gilet L, Figaro S, Hammann P, Kuhn L, et al. tRNA maturation defects lead to inhibition of rRNA processing via synthesis of pppGpp. Mol Cell. 2019; 74: 1227-1238.
9. Kriel A, Bittner AN, Kim SH, Liu K, Tehranchi AK, Zou WY, et al. Direct regulation of GTP homeostasis by (p) ppGpp: a critical component of viability and stress resistance. Mol Cell. 2012; 48: 231-241.
10. Arnaud M, Chastanet A, Débarbouillé M. New vector for efficient allelic replacement in naturally nontransformable, low-GC-content, Gram-positive bacteria. Appl Environ Microbiol. 2004; 70: 6887-6891.
11. Guérout-Fleury A-M, Frandsen N, Stragier P. Plasmids for ectopic integration in Bacillus subtilis. Gene. 1996; 180: 57-61.
12. Bhavsar AP, Zhao X, Brown ED. Development and Characterization of a Xylose-Dependent System for Expression of Cloned Genes in Bacillus subtilis: Conditional Complementation of a Teichoic Acid Mutant. Appl Environ Microbiol. 2001; 67: 403-410.
13. Mirouze N, Prepiak P, Dubnau D. Fluctuations in spo0A transcription control rare developmental transitions in Bacillus subtilis. PLoS Genet. 2011; 7: e1002048.
